# Evidence for Glacial Refugia of the Forest Understorey Species *Helleborus niger* (Ranunculaceae) in the Southern as Well as in the Northern Limestone Alps

**DOI:** 10.3389/fpls.2021.683043

**Published:** 2021-05-10

**Authors:** Eliška Záveská, Philipp Kirschner, Božo Frajman, Johannes Wessely, Wolfgang Willner, Andreas Gattringer, Karl Hülber, Desanka Lazić, Christoph Dobeš, Peter Schönswetter

**Affiliations:** ^1^Department of Botany, University of Innsbruck, Innsbruck, Austria; ^2^Institute of Botany of the Czech Academy of Sciences, Průhonice, Czechia; ^3^Department of Botany and Biodiversity Research, University of Vienna, Vienna, Austria; ^4^Department of Forest Genetics and Forest Tree Breeding, Georg-August University of Göttingen, Göttingen, Germany; ^5^Institute of Forest Genetics, Austrian Research Centre for Forests, Vienna, Austria

**Keywords:** Alps, demographic modeling, forest understorey, glacial refugia, *Helleborus niger*, RAD sequencing, species distribution modeling

## Abstract

Glacial refugia of alpine and subnival biota have been intensively studied in the European Alps but the fate of forests and their understory species in that area remains largely unclear. In order to fill this gap, we aimed at disentangling the spatiotemporal diversification of disjunctly distributed black hellebore *Helleborus niger* (Ranunculaceae). We applied a set of phylogeographic analyses based on restriction-site associated DNA sequencing (RADseq) data and plastid DNA sequences to a range-wide sampling of populations. These analyses were supplemented with species distribution models generated for the present and the Last Glacial Maximum (LGM). We used exploratory analyses to delimit genomically coherent groups and then employed demographic modeling to reconstruct the history of these groups. We uncovered a deep split between two major genetic groups with western and eastern distribution within the Southern Limestone Alps, likely reflecting divergent evolution since the mid-Pleistocene in two glacial refugia situated along the unglaciated southern margin of the Alps. Long-term presence in the Southern Limestone Alps is also supported by high numbers of private alleles, elevated levels of nucleotide diversity and the species’ modeled distribution at the LGM. The deep genetic divergence, however, is not reflected in leaf shape variation, suggesting that the morphological discrimination of genetically divergent entities within *H. niger* is questionable. At a shallower level, populations from the Northern Limestone Alps are differentiated from those in the Southern Limestone Alps in both RADseq and plastid DNA data sets, reflecting the North-South disjunction within the Eastern Alps. The underlying split was dated to ca. 0.1 mya, which is well before the LGM. In the same line, explicit tests of demographic models consistently rejected the hypothesis that the partial distribution area in the Northern Limestone Alps is the result of postglacial colonization. Taken together, our results strongly support that forest understory species such as *H. niger* have survived the LGM in refugia situated along the southern, but also along the northern or northeastern periphery of the Alps. Being a slow migrator, the species has likely survived repeated glacial-interglacial circles in distributional stasis while the composition of the tree canopy changed in the meanwhile.

## Introduction

The climatic changes during the Quaternary have had a major impact on the distribution of many plant and animal species by inducing shifts, expansions, contractions, and fragmentations of ranges as well as population extirpations (Comes and Kadereit, 1998; Hewitt, 2001; Petit et al., 2003). In extensively glaciated mountain ranges such as the European Alps, species were forced into refugia during cold periods, from where they (re)colonized deglaciated areas during warmer periods including the Holocene (Schönswetter et al., 2005). Although the Quaternary climatic oscillations are expected to translate into cyclical contractions and expansions of species ranges, many species were not able to expand their ranges in times of suitable climatic conditions as is evident, for instance, from the incomplete range-filling of European temperate tree species (Svenning and Skov, 2007) and mountain plants of the Alps (Dullinger et al., 2012a).

The Eastern Alps have a clearly tripartite tectonic structure, with the siliceous Central Eastern Alps flanked by the calcareous Northern and Southern Limestone Alps (Froitzheim et al., 2008). During the Last Glacial Maximum (LGM), most of the Eastern Alps was strongly glaciated and only small areas along the southern, eastern, and northeastern Alpine periphery remained ice-free (Ivy-Ochs et al., 2008). The particular tectonic structure confers that calcicolous species, which are distributed in both the Northern and the Southern Limestone Alps, were either present in refugia adjacent to or overlapping with both areas during the last glacial period (Merxmüller, 1952, 1953, 1954; Tribsch and Schönswetter, 2003), or colonized one area from the other after the LGM (Schneeweiss and Schönswetter, 2010). Large-scale genetic exchange between both areas has likely been limited because of the scarcity of suitable substrate in the intervening siliceous Central Eastern Alps that acted as a strong barrier; in addition, any hypothetical populations in the Central Eastern Alps were extirpated during cold stages due to the area’s extensive glaciation (Ivy-Ochs et al., 2008).

Glacial refugia of biota and their consequences for species distributions have been intensively studied in the Alps, albeit with a strong focus on species of the alpine and subnival vegetation belts (Stehlik et al., 2002a; Schönswetter et al., 2005; Schneeweiss and Schönswetter, 2010). For instance, peripheral refugia in the Northern Limestone Alps have so far been identified for high-elevation species (Merxmüller, 1952, 1953, 1954; Stehlik et al., 2002b; Paun et al., 2008; Winkler et al., 2010), while the fate of forest understory species in that area remains largely unclear. To date, among the studies including samples from the area only Daneck et al. (2016) suggested that a glacial refugium in the eastern part of the Northern Limestone Alps “cannot be excluded” for *Rosa pendulina* based on somewhat ambiguous Amplified Fragment Length Polymorphism evidence. In contrast, a refugium in the southeastern Alps and the adjacent Balkan Peninsula was strongly supported, for instance, for *Cyclamen purpurascens* (Slovák et al., 2012) and *Hacquetia epipactis* (Urbaniak et al., 2018).

Paleoenvironmental reconstructions support the persistence of coniferous trees throughout the LGM at the south-eastern fringe of the Alps (van der Knaap et al., 2005; Monegato et al., 2015). According to these findings, Swiss stone pine (*Pinus cembra*), larch (*Larix decidua*), and dwarf mountain pine (*Pinus mugo*) formed a vegetation belt between alpine and lowland grasslands; in addition, spruce (*Picea abies*) and Scots pine (*Pinus sylvestris*) occurred in the alluvial plains. Mesophytic broad-leaved forest trees, such as beech (*Fagus sylvatica*), were absent from the Alpine fringe, but survived in refugia further south, in particular in the northwestern Balkan Peninsula (Magri et al., 2006). Pollen data as well as retrospective evaluation of current microclimatic variability also suggest an isolated microrefuge of broad-leaved trees in the Colli Euganei, close to the southern margin of the Alps (Kaltenrieder et al., 2009; Gubler et al., 2018). Paleontological data from the northeastern fringe of the Alps are still scarce, but broad-scale reconstructions suggest that the vegetation pattern was probably similar to that in the southeastern Alps (Willis and van Andel, 2004).

**Figure 1:**
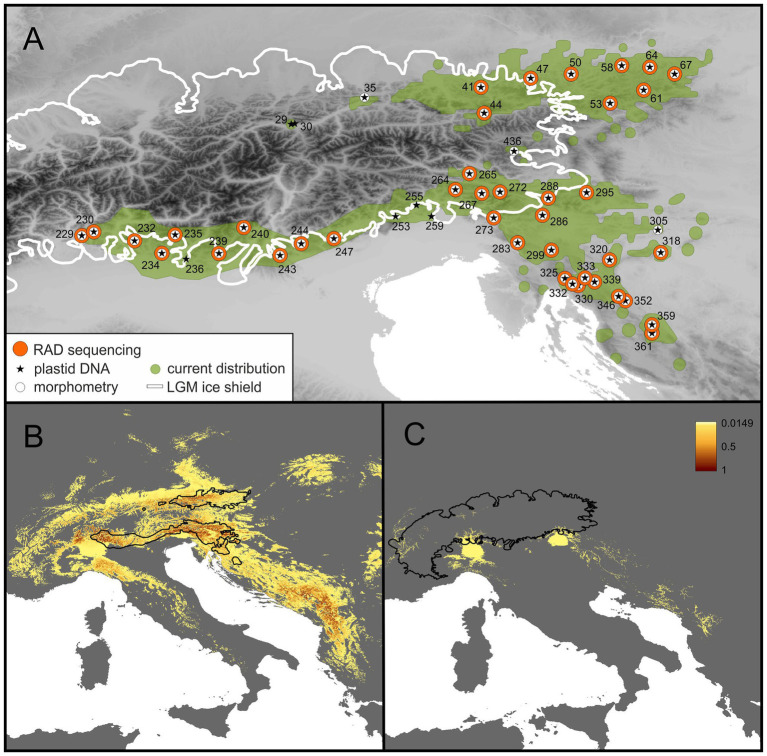
Current distribution and sampled populations of *H. niger* **(A)** as well as the potential distribution at present **(B)** and at the Last Glacial Maximum (LGM; **C**). **(A)** Current distribution (green), extent of glaciation during the LGM (white line) and sampled populations (numbered). Symbols indicate if a population was used for RADseq (orange dots), plastid DNA sequencing (stars), or morphometry (white dots). Locality information is detailed in Supplementary Table 1. **(B,C)** The occurrence probability derived from species distribution models (SDMs) is indicated by a color shade ramp; the terrestrial surface with occurrence probability <0.05 is in gray. In **(B,C)** the current distribution and the LGM glaciation of the Alps, respectively, are indicated by black lines.

Dominant European forest tree species are wind-pollinated, rendering the resolution obtained by phylogeographic studies relatively coarse (e.g., Taberlet et al., 1998; Tóth et al., 2017). Here, we aim to fill this gap by focusing on an insect-pollinated, ant-dispersed forest understory plant, *Helleborus niger* (Ranunculaceae). It is a herbaceous perennial with a robust rhizome and the only representative of *H.* section *Helleborus* (Ranunculaceae; Mattews, 1989; Sun et al., 2001). Chromosome counts (2*n* = 32) were reported, among others, from Italy (D’Amato and Bianchi, 1989) and Slovenia (Löve, 1971; Stace, 1995). It has an estimated genome size (2C-value) of 28.0–29.5 pg (Zonneveld, 2001; Temsch et al., 2010). Seeds are 3.5–5.5 mm large, with an elaiosome. Reproduction is sexual *via* seeds; limited clonal growth is possible *via* short rhizomes. Plants flower from December to March. The species is entomophilic and myrmecochorous (Chrtková, 1997; Klotz et al., 2002). Information on the generation times of *H. niger* is scarce, and the available evidence is mostly anecdotal, suggesting that it may take individuals between 4 and 10 years to flower and that genets are very long-lived due to their ability of clonal growth of the guerilla type (P. Schönswetter, W. McLewin, field observations; Silvertown and Charlesworth, 2009). *Helleborus niger* is distributed in the Northern Limestone Alps of Austria and adjacent Germany, and in the Southern Limestone Alps from the Swiss Ticino over Italy to Slovenia; from there, the range extends southwards to northern Croatia (Figure 1A; Meusel et al., 1965). The two partial distribution areas are separated by the predominantly siliceous Central Eastern Alps, where only a few isolated populations occur on islands of calcareous bedrock.

*Helleborus niger* occurs from the submontane to the high montane (rarely subalpine) vegetation belt (90–1,900 m a.s.l.) with a strong preference for carbonate bedrock (Fischer et al., 2008) and base-rich soils (Landolt, 1977; Ellenberg and Leuschner, 2010). The species shows a high affinity to forests dominated by beech; however, it also occurs in spruce, larch, and pine (*Pinus nigra*, *Pinus sylvestris*, *Pinus mugo*, *Pinus cembra*) forests or krummholz (Willner and Grabherr, 2007). It is sometimes split into two subspecies, the widespread type subspecies and the predominantly Southern Alpine *H. niger* subsp. *macranthus* (Freyn) Schiffn. (Schiffner, 1889). Currently, the latter subspecies is assumed to occur in the middle and western Southern Limestone Alps (e.g., Aeschimann et al., 2004) and in Croatia (Raab-Straube et al., 2014). The limitation to calcareous bedrock restricts its distribution to the Northern and Southern Limestone Alps and a few calcareous islands in the Central Eastern Alps. From the southeastern Alps, the species’ distribution extends to the northwestern Balkan Peninsula (Meusel et al., 1965; Figure 1).

**Figure 2:**
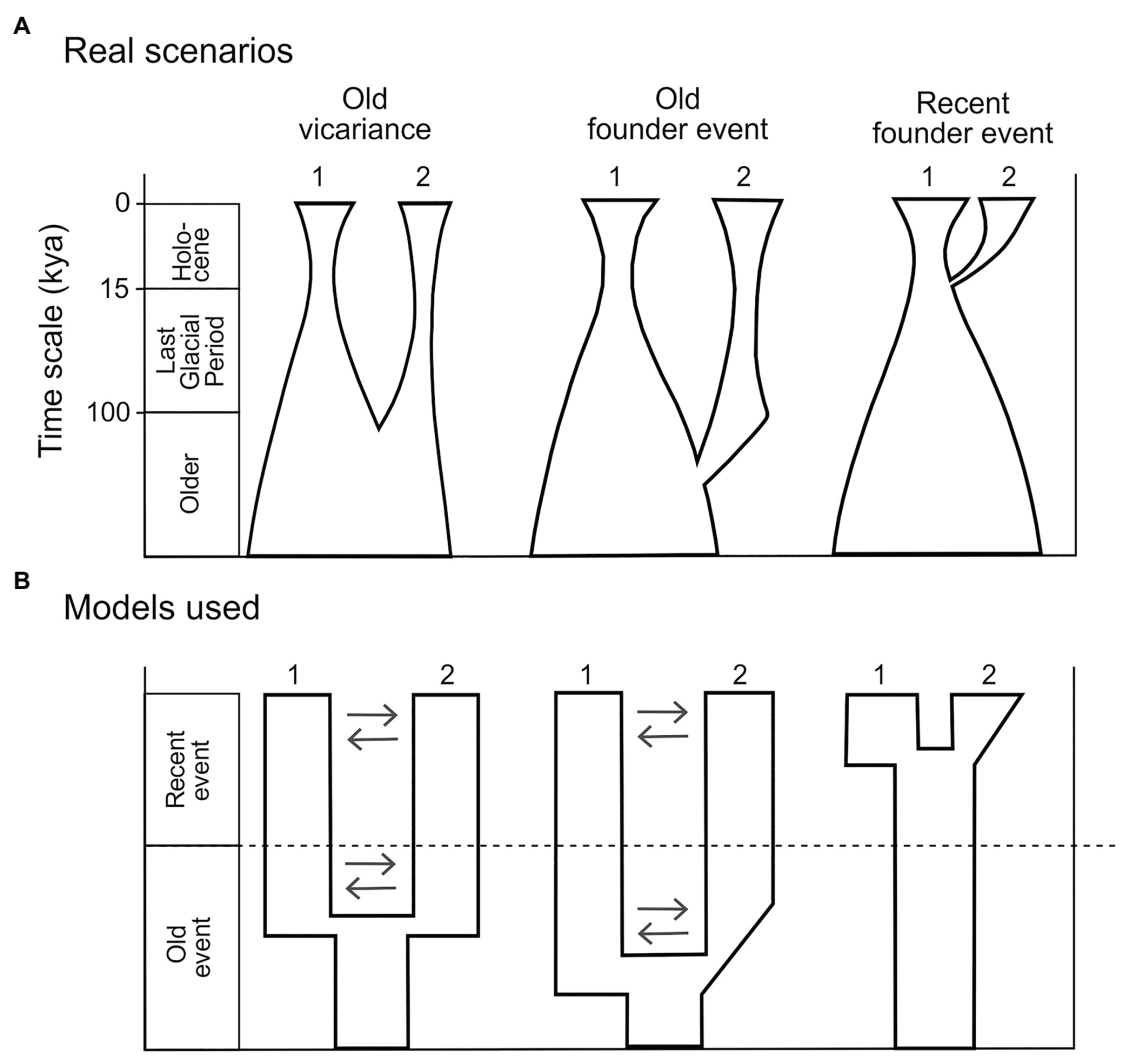
**(A)** Scenarios explaining the disjunct distribution of current *H. niger* populations. In the left and middle pane, pre-glacial vicariance or a pre-glacial founder event led to the split of the ancestral population into the current populations 1 and 2. In the right pane, a post-glacial founder event gave rise to population 2 from the ancestral population 1. See detailed description in the Introduction section. **(B)** Summary of models simplifying particular scenarios in **(A)**, tested using demographic models in dadi. All models tested are shown and described in Supplementary Figure 1. Numbers 1 and 2 refer to current populations (i.e., genetic groups defined based on STRUCTURE analysis); gene flow is indicated by arrows.

For a limestone-dwelling forest understory species such as *H. niger*, two particular scenarios may explain the current disjunct distribution in the Eastern Alps. (1) The Northern Limestone Alps were colonized postglacially from a single cold stage refugium in the weakly glaciated Southern Limestone Alps and/or adjacent areas in the northwestern Balkan Peninsula (Figure 2A, Recent founder event scenario). Under this scenario, the split between the populations in the Northern and the Southern Limestone Alps should postdate the LGM. Alongside demographic evidence such as a recent bottleneck followed by exponential population growth, we expect lower levels of genetic diversity and private alleles in the northern populations. (2) Cold stage refugia were available in both the Southern and the Northern Limestone Alps. This scenario implies that the split between the southern and the northern populations predates the LGM and that exponential population growth occurred before the last glacial period. The levels of genetic diversity and private alleles of the populations in the Northern Limestone Alps may differ depending on their origin. Specifically, vicariance (Figure 2A, Old vicariance scenario) is expected to have conferred a less pronounced reduction of genetic diversity than stepping-stone or long distance dispersal (Figure 2A, Old founder event scenario; e.g., Avise, 2000; Kropf et al., 2006).

The present study aims at shedding light on the underexplored biogeographic history of the forest vegetation of the European Alps by disentangling the spatiotemporal diversification of the disjunctly distributed, frequent, and abundant forest understory plant *Helleborus niger*, the black hellebore. First, we explored if the distribution of this presumably slowly dispersed species (Klotz et al., 2002) is delimited by its climatic niche or if the potential distribution markedly surpasses the actual one, indicating incomplete range filling (Svenning and Skov, 2004). Then, we applied a set of phylogeographic analyses based on restriction-site associated DNA sequencing (RADseq) data and plastid DNA sequences to a range-wide sampling of populations. We used exploratory analyses to delimit genomically coherent groups and then employed demographic modeling to reconstruct the history of these groups. First, we aimed to unravel if the disjunction between the Southern and the Northern Limestone Alps can be explained by glacial survival in both areas, or if the partial distribution area in the latter is the result of postglacial colonization. We did so by employing genomic and genetic data as well as a retrospective species distribution model. Further, we tested if there is evidence for gene flow between populations from the Southern and the Northern Limestone Alps or if both population groups represent isolated genetic groups. As we found a deep phylogeographic split within the Southern Limestone Alps, we applied the approaches outlined above to explore the diversification process within that area as well. Finally, we tested if the major genetic groups are reflected by differences in leaf shape (flowers and fruits were not available during the collecting season).

## Materials and Methods

### Plant Material

Forty-seven populations were visited in 2017 to sample young and healthy leaves of two to five (usually three) individuals per population for the DNA extraction (Supplementary Table 1). In 2019, two populations from the northwesternmost part of the distribution area were added to the sampling, that is, a newly found presumably native population (population 29) from a remote gorge, and a long-time known, likely planted population (population 30). Sampled individuals were separated at least 5 m from each other to avoid sampling clones. Based on our field experience, we are convinced that such a distance is sufficient, as the extent of spatially isolated individuals never surpassed one square meter. From each population, a herbarium voucher was made and deposited in the herbarium of the University of Innsbruck (acronym IB).^1^ Sampling was carried out in accordance with the Nagoya Protocol, and the EU regulation 511/2014, i.e., prior informed consent was requested from the competent national authorities of Nagoya protocol members^2^ and a collection permission obtained if required. *Helleborus foetidus* L. was sampled as outgroup.

### DNA Extraction

DNA was extracted from silica gel dried leaf material using the innuPREP Plant DNA Kit II (Analytik Jena, Jena, Germany). Dry leaf material (100 mg) was transferred together with two 4 mm steel balls into a 2 ml reaction tube and frozen for 10 min in liquid nitrogen. The material was immediately ground using a Retsch (Düsseldorf, Germany) swing mill for 4 min. Subsequently, 500 μl lysis solution SLS and 20 μl Proteinase K were added and mixed vigorously by pulsed vortexing for 5 s. During lysis, tubes were incubated at 65°C for 30 min. Lysis was stopped by addition of 100 μl precipitation buffer P and samples were vortexed again for 5 s, followed by incubation at room temperature for 5 min and centrifugation at 10,000 *g* for 5 min. The clear supernatant was transferred onto a prefilter and centrifuged at 10,000 *g* for 1 min. Cleaning of the extract by binding the DNA to magnetic beads, repeated ethanol precipitation and recovery of DNA was carried out by an InnuPure C16 robot (Analytik Jena). RNA was digested by adding 4 μl RNase A solution (100 mg/ml), followed by incubation for 5 min at room temperature. The quality of DNA was checked on an agarose gel. The DNA concentration was estimated using a Qubit 4 fluorometer (ThermoFisher Scientific, Darmstadt, Germany).

### RADseq: Library Preparation, Identification of RADseq Loci and SNP Calling

Single-digest RADseq libraries were prepared from three individuals from each of 40 populations of *H. niger* (with exception of populations no. 50 and 244 with two individuals sampled), and three individuals from two populations of *H. foetidus* as outgroup, using the restriction enzyme PstI (New England Biolabs) and a protocol adapted from Paun et al. (2016). Briefly, we started with 110 ng DNA per individual and ligated 100 mM P1 adapters to the restricted samples. Shearing by sonication was performed with a M220 Focused-ultrasonicator (Covaris) with settings targeting a size range of 200–800 bp and a mode at 400 bp (peak in power: 50, duty factor 10%, 200 cycles per burst and treatment time 90 s at 20°C). Libraries were sequenced on Illumina HiSeq at VBCF NGS Unit as 100 bp single-end reads.^3^

The raw reads were quality filtered and demultiplexed based on individual-specific barcodes using Picard BamIndexDecoder included in the Picard Illumina2bam package (available from https://github.com/wtsinpg/illumina2bam) and the program process_radtags.pl implemented in Stacks v. 1.35 (Catchen et al., 2011, 2013). The RADseq loci were further assembled, and single nucleotide polymorphisms (SNPs) were called using the “denovo_ map.pl” pipeline also implemented in Stacks. *Denovo*_map.pl was run on subsets of the raw data to infer the parameters for an optimal loci yield following Paris et al. (2017). Consequently, a dataset used for subsequent phylogenetic reconstruction was built using a minimum coverage to identify a stack of 6× (−m 6), a maximum number of differences between two stacks in a locus in each sample of five (−M 5), and a maximum number of differences among loci to be considered as orthologous across multiple samples of five (−n 5). The function “export_sql.pl” in the Stacks package was used to extract locus information from the catalog, filtering for a maximum number of missing samples per locus of 75% and a maximum number of SNPs per locus of 10. Furthermore, we filtered out loci if (1) in any of the samples more than two alleles were detected, to reduce the risk of including paralogs in the dataset, or (2) if the number of deleveraged tags was higher than 0. Final filtering for paralogs was done using PMERGE (Nadukkalam Ravindran et al., 2018); however, no additional paralogous fragments were identified by the software.

The program populations implemented in the software Stacks v. 1.35 was used to export the selected loci; whitelists were used to exclude unwanted loci as described above. For the first dataset, later used for Bayesian clustering, a set of RADseq loci was exported into STRUCTURE and vcf formats; to further filter missing data, we used -p 20 and -r 0.5 flags (minimum of 20 out of 40 populations present with at least 50% of individuals from each population) and finally the --write_single_snp flag was used to select only a single (first) SNP per fragment, to minimize the chance of selecting linked loci. For this dataset, all individuals sampled from the same locality were defined as a population. For the second dataset, later used for phylogenetic tree reconstruction, three individuals of *H. foetidus* were included in the popmap as outgroup, and -p and -r flags were omitted to obtain more variable sites. Finally, for preparation of site frequency spectra (SFS) for demographic modeling, the 10 individuals with the lowest proportion of missing data were selected from each STRUCTURE group (see below). For those individuals, SNPs were exported in vcf format with the program populations and further filtered using VCFtools 0.1.15 (Danecek et al., 2011) for a maximum of 50% of missing data and a minimum depth of coverage of 5×.

### Exploratory Analyses of SNP Data

For the *H. niger* samples (118 individuals), the optimal grouping of populations was determined using Bayesian clustering in STRUCTURE 2.3.4, using the admixture model with uncorrelated allele frequencies (Pritchard et al., 2000). Ten replicate runs for *K* (number of groups) ranging from 1 to 10 were carried out using a burn-in of 10,000 MCMC iterations followed by 100,000 additional iterations. The optimal *K* was identified as the *K*, where the increase in likelihood started to flatten out, the results of replicate runs were similar, and the clusters were non-empty. Additionally, the deltaK criterion was employed, reflecting an abrupt change in likelihood of runs at different *K* (Evanno et al., 2005). To estimate the number of private alleles and the nucleotide diversity (π) per population, we used the program populations in Stacks. The significance of pairwise differences in distributions between groups was estimated with Mann-Whitney tests using Past3 (Hammer et al., 2001).

To infer phylogenetic relationships among all 121 individuals (*H. niger* and *H. foetidus*), we computed a maximum likelihood (ML) phylogeny using RAxML v. 8.2.8 (Stamatakis, 2014). Invariant sites were removed from the original phylip format using the script “deleteAlignColumn.pl” (available from https://www.biostars.org/p/55555/) and Felsenstein’s ascertainment bias correction was further used to account for missing invariant sites (Leaché et al., 2015). Tree searches were done using the Jukes-Cantor substitution model (option -m ASC_GTRCAT --JC 69 -asc-corr=felsenstein; Stamatakis, 2014). The best-scoring ML tree was bootstrapped using 1,000 replicates and the frequency-based stopping criterion (Pattengale et al., 2010).

### Demographic Modeling

To investigate alternative divergence scenarios for the two main groups and two subgroups detected by STRUCTURE, we used the diffusion approximation method of dadi to analyze two-dimensional joint site frequency spectra (2D-JSFS, Gutenkunst et al., 2009). We used an established 2D analysis pipeline (Portik et al., 2017; Charles et al., 2018) and adapted publicly available python scripts^4^ that define 2D models, perform model fitting, and execute plotting functions.

The 2D-JSFS were prepared *via* conversion of a vcf file to a folded SFS. This was done by down-projection of the sampling size to maximize the number of sampled individuals while minimizing levels of missing data for downstream multi-population comparisons using easySFS.^5^ The down-projection resulted in the following numbers of alleles for the STRUCTURE groups and subgroups presented in the Results section: Western Group, 12 alleles; Eastern Group, 14 alleles; South-Eastern Subgroup, 12 alleles; and North-Eastern Subgroup, 12 alleles.

The 2D analysis pipeline was applied to pairwise comparisons between (i) Western Group vs. Eastern Group and (ii) South-Eastern Subgroup vs. North-Eastern Subgroup. For both comparisons, we translated three scenarios (Figure 2A) into three demographic models (Figure 2B). Specifically, we tested whether one of the genetic groups is the result of postglacial expansion from the other group (Scenario “Recent founder event”; Figure 2A). Alternatively, both genetic groups may have survived at least the last glacial period in separate refugia. Accordingly, we defined two additional scenarios, contrasting the “Recent founder event” scenario: In the first scenario, the observed divergence pattern is the result of old vicariance (scenario “Old vicariance”; Figure 2A). In the second scenario, we hypothesize that one of the groups (i.e., the North-Eastern subgroup) originated *via* an old (i.e., non-postglacial) founder event (scenario “Old founder event”; Figure 2A). Under these two scenarios, the latter group experienced at least one bottleneck due to the reduction of potential habitat caused by the expansion of glaciers. This should be reflected in a decrease of effective population size during the LGM, and followed by population expansion during the Holocene. However, detection of exponential growth in the last 10,000 years is challenging, especially for organisms with long generation time (>10 years; Adams and Hudson, 2004; Elleouet and Aitken, 2018). Therefore, we chose to test the simplified models, including either (i) no change in population size since the split, (ii) an early stage of population expansion followed by constant population size, or (iii) a continuously expanding population size (Figure 2B). In the context of demographic modeling we refer to the genetic groups identified by the STRUCTURE analyses as “populations” in accordance with recent studies (e.g., Portik et al., 2017; Charles et al., 2018).

To differentiate the scenarios of a pre-glacial vs. a post-glacial split of the populations, we dated the splits between groups using the Bayesian program BPP (see below). For both pairwise comparisons, we tested eight island demographic models (“island” referring to the derived, recently founded population in contrast to the ancestral “mainland” population; we use the more intuitive terms “derived population” and “ancestral population” in the following). These eight models represent the three main hypotheses and modifications with ancestral or recent migration included to explain additional features of the 2D-JSFS (Supplementary Figure 1). While the models representing old vicariance and a recent founder event were taken from Charles et al. (2018), the models including an old founder event were newly designed here. For models in both the vicariance and founder event categories, we followed the strategy of Charles et al. (2018) and included a variable *s* that defines the fraction of the ancestral population (nuA) founding each daughter population, where nuA*s represents the island population and nuA*(1−s) represents the mainland population. We enforced an upper limit of 0.5 for *s*.

In the optimization of the models, we initially optimized 3-fold perturbed random starting parameters followed by two rounds of 2-fold and one round of 1-fold perturbed parameters to estimate the log-likelihood of the SFS given the model. The number of replicates in each round was 10, 20, 30, and 60, respectively. Across all analyses, we used the optimized parameter sets of each replicate to simulate the 2D-JSFS, and a multinomial approach to estimate the log-likelihood of the 2D-JSFS given the model. The models were compared using the Akaike information criterion (AIC), and the replicate with the highest likelihood for each model was used to calculate AIC scores, ∆AIC scores, and Akaike weights (ω_i_; Burnham and Anderson, 2002).

We did not transform parameters into biologically meaningful estimates because our primary aim was to differentiate among different demographic scenarios *via* model selection (Gutenkunst et al., 2009). Instead, we estimated times of the splits modeled above using the BPP approach applying the range of potential mutation rates and generation times described below.

### Dating of the Phylogenetic Tree

**Figure 3:**
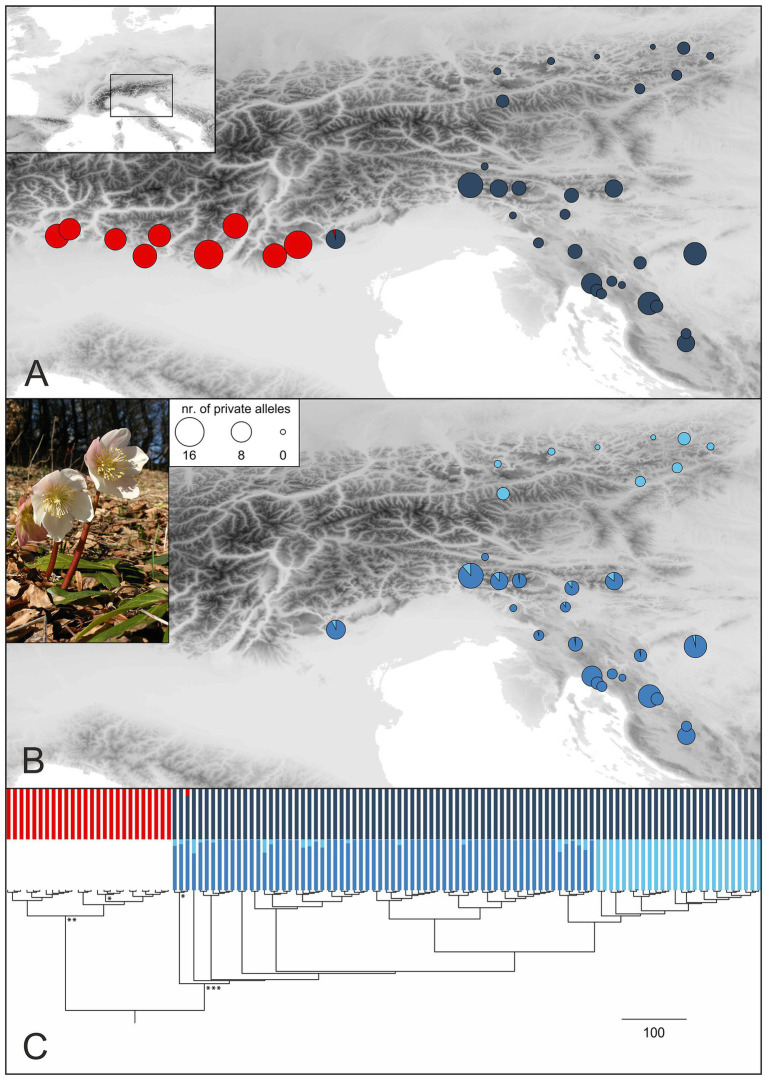
Genetic structure in *H. niger* (pictured) based on RADseq. **(A)** STRUCTURE analysis of the entire RADseq dataset yielded two groups (cluster memberships were summarized for all analyzed individuals per population; red, Western Group; blue, Eastern Group) as optimal solution. Sizes of pie charts reflect the number of private alleles in each population. **(B)** Subclusters (middle blue, South-Eastern Subgroup; light blue, North-Eastern Subgroup) as identified by STRUCTURE analysis of the Eastern Group. **(C)** Phylogenetic analysis conducted with RaxML; the outgroup *Helleborus foetidus* is not shown. Bootstrap support (BS) of major clades is indicated by asterisks (*, 74% BS; **, 87% BS; ***, 96% BS); a tree with labeled terminals and bootstrap support values is presented in Supplementary Figure 5. The tree is complemented with barplot representations of the STRUCTURE results shown in **(A,B)**.

The relative age of major phylogenetic splits was inferred under a multi species coalescent model as implemented in BPP (Flouri et al., 2018). Time estimates were done using the fixed topology (option A00 in BPP) resulting from the maximum likelihood based phylogeny (Figure 3C). More specifically, the node age of the split between the Western Group and the Eastern Group, and the node age of the split between the South-Eastern and the North-Eastern Subgroups were estimated. To do so, all RADseq tags were exported from the Stacks catalogue using populations.pl and the --fasta-samples flag (Catchen et al., 2013). The fasta file was converted to phylip format using the python script fasta2genotype.py (Maier et al., 2019), and by doing so, also loci with excess heterozygosity (>65%) were removed to filter for paralogs. For further analysis, the individuals with the lowest amount of missing data were selected from each of the three clusters, resulting in an alignment with 29 individuals (Western Group 10, South-Eastern Subgroup 9, and North-Eastern Subgroup 10). The R package phrynomics (Banbury and Leaché, 2014) was used to filter RADseq tags that were missing in >40% of the samples, which resulted in a final alignment containing 900 RADseq tags. Due to the large computational demand in estimating node ages in BPP, five alignments containing random subsets of 100, 200, 300, 400, and 500 RADseq tags were created for analysis. This was done to later evaluate the consistency of node age estimates. Calculations in BPP were done using default settings and assuming that the data are diploid and unphased (Flouri et al., 2018). MCMC chains were run for 1,000,000 generations, and after discarding 10% of the MCMC as burnin, Tracer (Rambaut et al., 2018) was used to check for convergence and to ensure that the effective sample size of all estimated parameters was >200.

To calibrate *τ* (τ = 2μt; *μ* = mutation rate per site per generation, *t* = divergence time) to absolute time, the function msc2time.r implemented in the R package bppr was used (Angelis and Dos Reis, 2015; Yoder et al., 2016). Briefly, this approach uses gamma densities to obtain random samples of *μ* (substitution rate per site per generation) and generation time from the “mcmc file” returned by BPP (Flouri et al., 2018). Based on these procedures, the substitution rate per time unit is calculated, and subsequently used to calculate absolute divergence times from *τ*. We used a *μ* value of 7 × 10^−9^ (Ossowski et al., 2010), and allowed a deviation of 10% from the above rate to account for uncertainty concerning the range of *μ* in herbaceous Angiosperms. As detailed information on the generation time of *H. niger* is lacking (see above), we chose generation times of 5, 10, and 20 years to represent a range of genealogical scenarios for the species. We emphasize that the demographic generation time is not the time until the first reproduction but the average time between two successive generations within a population (Charlesworth, 1994).

### Amplification and Sequencing of Plastid DNA Sequences, Analysis of Sequence Data

Three plastid DNA regions totaling 6,800 bp, that is, *ndhJ*–*trnT*, *rpoB–psbM*, and *trnQ*–*trnK* (Shaw et al., 2005, 2007) were inspected for variability. The *rpoB–psbM* region was most variable and was thus amplified for one individual per population from 50 populations of *H. niger* as well as for one individual of the outgroup *H. foetidus*. Amplification was performed in a reaction mix (total volume 31 μl) containing 11.6 μl of RedTaq ReadyMix (Sigma-Aldrich), 1.5 μl of BSA (1 mg/ml; Promega), 0.8 μl of each primer (10 μM) rpoB and psbMR (Shaw et al., 2005), and 1 μl of template DNA. Cycling conditions were 5 min at 94°C, 35 cycles of 1 min at 95°C, 1 min at 50°C, and 4 min at 65°C, followed by 5 min at 65°C. PCR programs were run on Eppendorf 5331 thermocyclers (PE Applied Biosystems, Foster City, CA, United States). PCR products were purified with *E. coli* Exonuclease I and shrimp alkaline phosphatase (SAP; Fermentas, St. Leon-Rot, Germany) following the manufacturer’s instructions. Sequencing was then carried out at Eurofins Genomics (Ebersberg, Germany) using the primers rpoB, trnC^GCA^F, and ycf6F (Shaw et al., 2005). Contigs were assembled, edited, and sequences aligned using Geneious Pro 5.5.9 (Kearse et al., 2012). The sequences obtained with the primers trnC^GCA^F and ycf6F were overlapping, thus including the *trnC*–*ycf6* spacer, the gene *ycf6*, and a part of the spacer *ycf6*–*psbM*. On the other hand, the sequences of the *rpoB*–*trnC* spacer obtained with the primer rpoB remained disconnected and we concatenated them with the *trnC*–*psbM* region. We constructed a statistical parsimony network of the concatenated alignment using TCS (Clement et al., 2000), with the connection limit set to 95. Gaps were treated as fifth character state and indels longer than 1 bp, which were only present in the outgroup, were reduced to single base pair columns allowing those structural mutations to be counted as single base pair mutations only.

### Morphometry

Morphometry was performed on rosette leaves of *H. niger* individuals from all populations studied with RADseq. Furthermore, we included populations 35, 305, and 436, for which only plastid sequences are available. One fully developed leaf per population was collected, pressed, and scanned at 400 dpi on a Konica Minolta c554 scanner (Konica Minolta, Marunouchi, Japan). Scans were saved as grayscale in bitmap format. The program tpsDig264 version 2.25 (Rohlf, 2016) was used to set landmarks at the very limits of the following characters: (A) central leaflet: (1) length and (2) maximum width of the blade, (3) length of the petiole, (4) distance of character 2 from the tip, (5) start of the dentation from the tip, (6) width and (7) side length of the terminal tooth, and length of the distal (8) and (9) proximal side of the tooth neighboring the terminal one; (B) lateral leaflet neighboring the central one: (10) length of the plate and (11) length of the petiole; (12) length of the petiole of the lowermost leaflet(s); and (13) length of the petiole supporting the lateral leaflets. Characters were derived from the landmarks by calculating the linear distance among the coordinates defining a character using basic geometrics programmed in a custom script. The following additional characters were manually scored: (14) number of teeth of the central leaflet (counted on one side) and (15) maximum angle of the side teeth relative to the border of the central leaflet.

Variables were scaled to zero mean and unit variance (i.e., z-transformed) to avoid effects of different value ranges. In order to remove size effects potentially exerted by the environment or the developmental stage of the leaves, we normalized the metric characters 1–13 by division through the sum of their values calculated for every individual. Therefore, subsequent analyses are solely based on leaf shape. The transformed variables were tested for normal distribution using the Shapiro-Wilk test (function *shapiro.test*). Non-normally distributed variables were transformed to normality using the *BoxCox* function of the library hdrcde (Hyndman et al., 2020). Correlation coefficients were computed for all pairwise combinations of variables using the *cor* function in order to detect undesirable high correlations (Pearson’s correlation coefficient *r* ≥ 0.90). We ran a principle component analysis (PCA) using the *eigen* function. A discriminant analysis (DA) was carried out to explore morphological separability of the two main genetic groups (Western Group and Eastern Group; see Results section) using the *lda* function from the *MASS* library (Venables and Ripley, 2002). Finally, we correlated the original variables with the canonical axis to explore their relative contribution to the separation (i.e., the canonical structure). All analyses were conducted in R 3.6.1 (R-Development-Core-Team, 2017).

### Environmental Variables

Because of the rough terrain of mountain regions within the study area, the coarse spatial resolution of available historic climate data sets does not allow reconstructing the migration history of species since the LGM. We, hence, applied a statistical downscaling procedure previously used in several studies on climate change effects (Zimmermann et al., 2009; Ramirez-Villegas and Jarvis, 2010; Tabor and Williams, 2010; Dullinger et al., 2012b). The “delta-method” uses deviations (i.e., deltas) between historic and current climatic conditions within predicted (i.e., hind-casted) time series, which are spatially interpolated from their coarser spatial resolution to those of current data series based on measurements using a cubic-spline algorithm and, finally, added to the latter. As historic data, we used reconstructions of the paleoclimate based on the Community Climate System Model ver. 3 (CCSM3) with a spatial resolution of 2.5°. This data emerged from the TRaCE21ka experiment (Liu et al., 2009; Otto-Bliesner et al., 2014) and are accessible *via* PaleoView (Fordham et al., 2017). Current climatic conditions with a spatial resolution of 30″ (i.e., approximately 1 km within the study area) were retrieved from the Chelsa Climate database (Karger et al., 2017a,b) available at http://chelsa-climate.org/. Due to the short temporal overlap between Chelsa data (representing averages of the years 1979–2013) and the paleoclimate (ending 1983), the delta-method was applied in a two-step approach including climate data having a temporal overlap of some decades between both data sets. We used the CRU TS4.01 data set (Harris et al., 2014) with a spatial resolution of 0.5° covering the period 1901–2016. As the first step, we calculated arithmetic means of climate variables (i.e., monthly precipitation as well as minimum, maximum, and average monthly temperature) of the paleoclimate data for two 30-year periods. These periods represent 17,100 BP, corresponding to a marked temperature minimum in the study area associated with Heinrich Stadial 1 (Hodell et al., 2017), and recent climatic conditions (i.e., 1950–1980). The latter is used as a reference period to enable the link to the CRU data. Further, we defined the deltas of climate variables as differences between historic and current (minimum, maximum, and average) temperature as well as the ratio (historic/current) in case of average precipitation. These deltas were computed between each of the three historic periods and the 1950–1980 reference period at 2.5° resolution, spatially interpolated to 0.5° to match the resolution of the CRU data using the thin plate spline method and added to the CRU data. This procedure was repeated in a second step of the downscaling approach to further link the modified CRU data (0.5°) to the Chelsa climatologies (30″) by calculating deltas of climate variables of the modified CRU data between the three historic periods and the reference period of 1979–2013, interpolating these deltas to the resolution of the Chelsa climatologies, and adding them to the Chelsa data. We used these downscaled monthly projections of climate variables to calculate four bioclimatic variables representing the amount and variability in precipitation and temperature: mean annual temperature (bio1), temperature seasonality (bio4), annual precipitation sum (bio12), and precipitation seasonality (bio15). These climatic variables were projected to a grid of 1 × 1 km cell size using the nearest neighbor method. As additional environmental variable, we used topographical roughness as the SD of the elevations of 100 × 100 m cells within each 1 × 1 km cell. These variables were checked to avoid high correlations (Pearson’s *r* > |0.7|).^6^

### Modeling Occurrence Probability

We used species distribution models (SDMs) to project the environmental suitability of the study area for *H. niger* at the end of the LGM and under current conditions. These models relate species occurrences to the five environmental variables described above at a resolution of 1 km. SDMs were based on generalized linear models (GLMs) starting with a niche determined using the climate of the current distribution of *H. niger*. However, this entailed a clearly too narrow species’ niche as the model predicted an extinction of the species, i.e., no cell within the study region was predicted to be suitable at 12,100 BP (representing the Younger Dryas, a particularly cold period after the LGM; see Supplementary Figure 2). This is in clear contradiction to the molecular evidence. Consequently, we added climatic conditions from potential refugial areas at the LGM as identified by a combination of STRUCTURE clusters (Figure 3A), LGM glaciation (Figure 1A), and genetic divergence (i.e., number of private alleles; Figure 3A). As a first step, sampled populations (see Plant Material section) were used as presences, while 10,000 pseudo-absences were randomly drawn across Europe but excluding the current range of *H. niger* (Figure 1A) following adapted recommendations by Barbet-Massin et al. (2012). These presence/absence data were used to fit a GLM including quadratic terms of all five environmental variables and to predict the occurrence probability of the study species at the two time steps mentioned above. In a further step, the climatic conditions of the cell with the highest occurrence probability for 17,100 BP in each refugial area were added as presence to the dataset. Pseudo-absences were generated by selecting 5,000 absences from each of the historic and current climate across Europe excluding refugia and glaciated areas at the LGM or the current distribution. A GLM was refitted to finally predict occurrence probabilities at each of the four time steps. Glaciated areas were defined as in Ehlers et al. (2011) supplemented by Zasadni and Klapyta (2014) for the Tatra mountains. Ice-free islands within the ice-shield of the Alps were removed.

## Results

### Population Structure and Phylogenetic Relationships

The average number of high-quality reads per sample retained after demultiplexing and quality filtering was 0.88 (*SD* = 0.16) million. The *denovo*_map.pl pipeline (ustacks) identified a mean coverage of 10.68× (±1.01) over all samples in the catalog (Catchen et al., 2013). All resulting raw RADseq data are available in the NCBI Short Read Archive as BioProject PRJNA716097 (accession numbers SRR14023579–SRR14023696, SRR14181234–SRR14181236, and SRR14181231–SRR14181233; details are given in Supplementary Table 1).

Bayesian population clustering analysis based on 1,074 unlinked SNPs (Supplementary Table 2) resulted in the detection of two allopatrically distributed genetic groups (Figure 3A; Supplementary Figure 3), the Western Group distributed in the western part of the Southern Limestone Alps and the Eastern Group distributed in the eastern parts of the Northern and Southern Limestone Alps, from where it extends to the northwestern Balkan Peninsula. The western-most population of the Eastern Group is slightly admixed. A STRUCTURE analysis failed to detect a substructure within the Western Group, but the Eastern Group was split into two subclusters termed North-Eastern Subgroup and South-Eastern Subgroup in the following (Figure 3B). Although these groups have allopatric distributions, traces of admixture were observed mainly in the northern-most populations of the South-Eastern Subgroup. For follow-up analyses, we thus defined three genetically and geographically distinct major phylogeographic groups. Although the total number of (both variable and invariant) sites recovered was comparable among the three groups (Table 1), the number of private alleles (Figures 3A,B) and the nucleotide diversity (π) per population differed significantly. The number of private alleles per population ranged between 9 and 16 for the Western Group, between 1 and 12 for the South-Eastern Subgroup, and between 0 and 3 for the North-Eastern Subgroup. Nucleotide diversity followed the pattern of the number of private alleles, with highest values observed in the Western Group (Table 1; Supplementary Figure 4; Supplementary Table 1).

**Table 1:** Descriptors of the population groups of *Helleborus niger* identified based on restriction-site associated DNA sequencing (RADseq) data generated.

	Sites recovered	Percentage of polymorphic loci	Expected heterozygosity	Nucleotide diversity (π)	Private alleles
Western Group	62834.4	24	0.0010	0.0013	11.9
Eastern Group	72742.5	19	0.0008	0.001	3.6
South-Eastern Subgroup	72698.0	20	0.0008	0.001	4.5
North-Eastern Subgroup	72851.3	17	0.0007	0.0009	1.4

Values are averaged over populations within each group.

A RAxML phylogeny based on 4,914 SNPs (Supplementary Table 2) that was rooted using *H. foetidus* as outgroup resolved *H. niger* as monophyletic (bootstrap support, BS, 89%; Supplementary Figure 5; Figure 3C). Within *H. niger*, two main clades were identified, corresponding to the Western and Eastern Groups (BS 87 and 96%, respectively). Relationships within the two clades were not supported (BS < 50%). However, the North-Eastern Subgroup was resolved as monophyletic sister-group to two northern populations of the South-Eastern Subgroup (264 and 267).

### Demographic History

**Figure 4:**
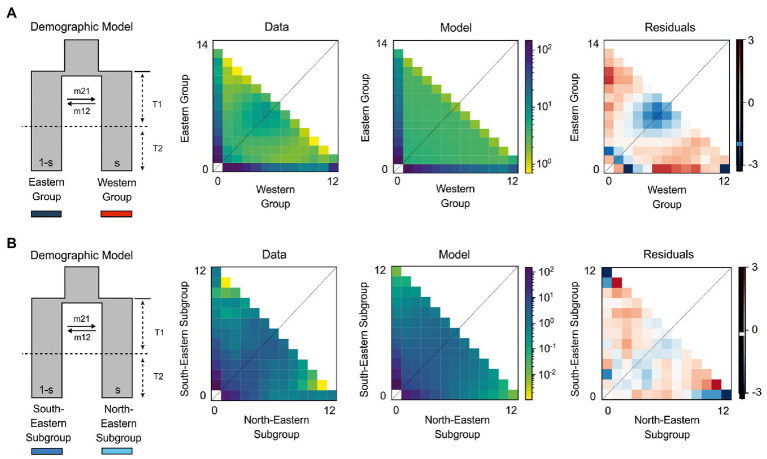
The best demographic models for *H. niger* using two-dimensional joint site frequency spectrum (2D-JSFS) between population sets including **(A)** Eastern (*n* = 14) and Western (*n* = 12) Group and **(B)** South-Eastern (*n* = 12) and North-Eastern Subgroup (*n* = 12). A visual representation of the best-fit model is depicted, along with comparisons of the 2D-JSFS for data, model, and resulting residuals. For both comparisons, the best-fit model represents the scenario where the ancestral population split due to vicariance. This was first followed by period T1 of continuous asymmetric migration between newly established populations, and then by period T2 of isolation. Additional models and parameter values are provided in Table 2 and Supplementary Table 3.

Detailed results of our demographic modeling are given in Table 2; Supplementary Table 3; Figure 4. For all models, the variation in log-likelihood scores observed across the initial optimizations of highly perturbed random starting parameters decreased during subsequent analyses incorporating less perturbed parameters from previous replicates, producing more consistent log-likelihoods.

**Table 2:** Two best demographic models and parameter values (unscaled) for pairwise population comparisons.

Population 1 vs. population 2	Model	Log-I	ω_i_	theta	nuA	nu1	nu2	T	T1	T2	*s*	m12	m21
Eastern vs. Western Group	vic_anc_asym_mig	−257.1	0.8	515.2	0.48	0.46	0.11		0.45	0.08	0.49	1.74	5.10
	vic_no_mig	−270	0.1	369.4	0.68	0.1	7.94	0.2			0.42		
South-Eastern vs. North-Eastern Subgroup	vic_anc_asym_mig	−139.8	0.5	123.8	2.36	0.29	0.36		0.99	0.05	0.27	6.77	0.21
	founder_anc_asym_two_epoch	−140.4	0.2	23.8	8.01	2.5	3.37		3.87	0.22	0.12	1.08	0.19

Detailed results of all models tested are provided in Supplementary Table 3. Vic_anc_asym_migr, vicariance with ancient continuous asymmetric migration followed by isolation; vic_no_mig, vicariance with no migration; vic_anc_asym_mig, vicariance with ancient continuous asymmetric migration followed by isolation; founder_anc_asym_two_epoch, old founder event with exponential size growth and continuous asymmetric migration followed by isolation; ω_i_, Akaike weight; theta (4N_ref_μL, where *L* is the total length of the sequenced region SNPs were ascertained from), the effective mutation rate of the reference population (here corresponds to the ancestral population; nuA, effective population size of ancestral population; nu1, nu2, effective population sizes of population 1 and 2, respectively, under the constant population size model; T, unscaled time of the population split; T1, unscaled time between the population split and the ancient migration; T2, unscaled time between the ancient migration and the present; *s*, fraction of nuA that goes to population 2; m12, migration rate from population 2 to population 1; m21, migration rate from population 1 to population 2.

In all comparisons, models of vicariance fitted better to our data than founder event models (Supplementary Table 3). We performed comparisons of Western and Eastern Groups with both groups acting either as ancestral population in founder event models or as major fraction of the ancestral population in case of vicariance models. The first two best models failed to identify whether the Western or the Eastern Group contributed the major fraction of the ancestral population (parameter *s*; Table 2). We found strong support (80% of the total model weight) for a vicariance model with ancient continuous asymmetric migration (ΔAIC = 4.16, ω_i_ = 0.8; Figure 4A). Migration was stronger from the Eastern Group to the Western Group (m_21_ = 5.1, m_12_ = 1.7). The second-best model (ΔAIC = 5.3, ω_i_ = 0.1; Supplementary Table 3) suggested vicariance without migration while the Western Group represented the major fraction (1−*s* = 0.58) of the ancestral population.

Similarly, in the pairwise comparison between the South-Eastern and the North-Eastern Subgroup, the best fitting model (ΔAIC = 1.28, ω_i_ = 0.51; Figure 4B) was a vicariance model with ancient continuous asymmetric migration (Table 2; Supplementary Table 3). The proportion of the ancestral founder population for the North-Eastern Subgroup (s) was estimated to be 0.27 and gene flow was inferred to be larger from the North-Eastern to the South-Eastern Subgroup (m_12_ = 6.7, m_21_ = 0.2; Table 2). The second-best model (ω_i_ = 0.2) included an old founder event with exponential population growth of the North-Eastern Subgroup and continuous asymmetric migration from the North-Eastern to the South-Eastern Subgroup followed by a period with stable population sizes.

### Divergence Time Estimation

The BPP analysis based on 500 RADseq tags estimated the phylogenetic splits between the Western and Eastern Groups, and between the South-Eastern and North-Eastern Subgroup at 6.2 × 10^−4^ and 1.7 × 10^−3^ *τ* units, respectively. The estimation of *τ* from BPP runs with fewer RADseq tags was consistent and yielded results in a similar range (Supplementary Table 4). Translating *τ* to absolute time, irrespective of the chosen generation time, inferred that both the split between the Western and the Eastern Group, and the split between the South-Eastern and the North-Eastern Subgroup, have occurred well before the LGM (Table 3). Using a short generation time of 5 years, the lower boundary of the 95% HPD is 0.3 mya for the split between the Western and the Eastern Group (mean 0.4 mya) and 48 kya for the split between the South-Eastern and the North-Eastern Subgroup (mean 85 kya).

**Table 3:** Divergence time estimates in million years for the phylogenetic splits identified within *H. niger*.

Split	Generation time in years	Mean	95% HPD lower	95% HPD upper
Eastern Group vs. Western Group	5	0.447	0.318	0.585
	10	0.894	0.640	1.173
	20	1.788	1.277	2.346
South-Eastern Subgroup vs. North-Eastern Subgroup	5	0.085	0.048	0.125
	10	0.165	0.095	0.249
	20	0.339	0.192	0.500

Divergence times estimates are based on a multispecies coalescent model using 500 randomly selected RADseq tags. Absolute times for each split were calculated for three different generation times.

### Plastid DNA Sequences

**Figure 5:**
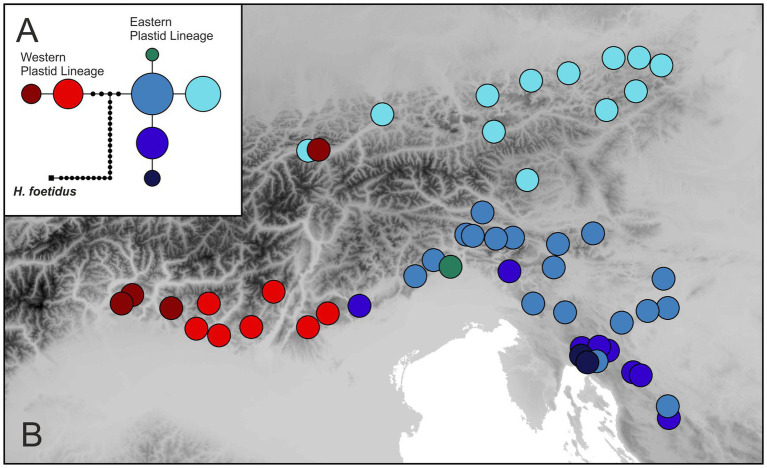
Plastid DNA variation in *H. niger* based on partial sequences of the *rpoB–psbM region*. **(A)** Statistical parsimony network including the outgroup species *H. foetidus*. The size of a circle is relative to the square root of a haplotype’s frequency. Haplotypes not sampled are shown as small black dots. **(B)** Distribution of haplotypes.

The concatenated alignment of *rpoB*–*trnC* and *trnC*–*psbM* sequences was 2,405 bp (2,377 bp after the exclusion of indels in the outgroup *H. foetidus*; Genbank accession numbers are given in Supplementary Table 1). Eight different haplotypes were revealed, the most divergent one being restricted to *H. foetidus*. The statistical parsimony network (Figure 5A) showed that the two main, geographically segregated haplotype lineages within *H. niger* are separated by five substitutions. The distribution of the Western Plastid Lineage is congruent with that of the Western Group retrieved from RADseq and additionally included population 30 from the Northern Limestone Alps not included in the RADseq data. It comprised two haplotypes (light and dark red in Figure 5) separated by one substitution; populations harboring these two haplotypes were geographically segregated. The Eastern Plastid Lineage included five haplotypes separated by one to two substitutions from the central haplotype. One haplotype (light blue in Figure 5) was geographically segregated, distributed in the Northern Limestone Alps and the Central Eastern Alps, and included all populations affiliated to the North-Eastern Subgroup identified based on RADseq data plus two populations from the western part of the Northern Limestone Alps not included in the RADseq data. The other haplotypes (light to dark blue and green) were more or less co-distributed in the southeastern Alps and the northwestern Balkan Peninsula. These haplotypes were only found in populations pertaining to the South-Eastern Subgroup.

### Morphometry

**Figure 6:**
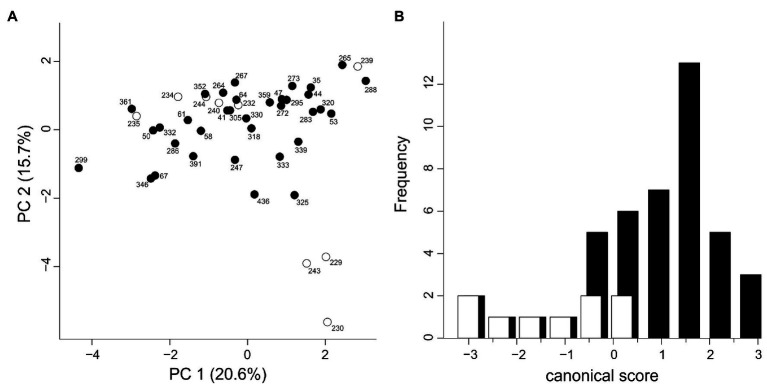
Morphological variation in *H. niger* based on 15 morphological characters describing the shape of rosette leaves; one individual each from 44 populations was investigated. White, Western Group; black, Eastern Group. **(A)** Principal component analysis. **(B)** Canonical discriminant analysis.

The measured morphological characters were all variable among individuals. No character had to be omitted because all pairwise Pearson correlation coefficients were below 0.90. The first two components of the PCA explained 36.3% of the total variance in the data. The PCA did not show obvious clustering of individuals by genetic affiliation although some individuals of the Western Group were separated from the bulk of individuals along the second principal component (Figure 6A). The characters contributing most strongly to the second principal component were characters 1 (loading: 0.500), 10 (0.410), and 4 (0.372). Likewise, the DA failed to show a clear separation between Western and Eastern Groups (Figure 6B). The classificatory DA classified only a moderate fraction of 44.4 and 74.3% (68.2% in total) of the individuals according to the prior assignment. The characters “maximum width of the blade,” “length of plate of lateral leaflet neighboring the central one,” and “length of the petiole supporting the lateral leaflet” showed the highest correlations with the canonical axis (Supplementary Table 5).

### Modeling Occurrence Probability

The SDM predictions for the current climatic conditions (Figure 1B) do not only fully cover the species’ present-day distribution, but rather clearly surpass it. Not occupied regions identified as climatically highly suitable are the southern Dinaric Mountains (Albania, Montenegro), the northernmost Apennines (Italy) and, to a lesser extent, the Western Carpathians (Slovakia, Poland). Climatic suitability of the study area for *H. niger* was generally much lower at the LGM (Figure 1C). However, the SDM indicates potential refugia in the Southern Limestone Alps within the two areas currently occupied by the Western Group and the South-Eastern Group. There was no support for a refugium in the eastern part of the Northern Limestone Alps, where the North-Eastern Subgroup is distributed. In addition, parts of the Dinaric Mountains, the northern Apennines, and the western French Alps were indicated as climatically suitable at the LGM.

## Discussion

Back hellebore, *H. niger*, is a frequent and often abundant calciphilous herbaceous perennial predominantly growing in the understory of beech forests, but extending also to other forest types (Willner and Grabherr, 2007; Supplementary Table 6). It is endemic to the Southern and Northern Limestone Alps as well as the adjacent northwestern Balkan Peninsula (Figure 1A). Species distribution modeling elucidated that the species’ present-day distribution is much smaller than the area representing its climatic niche (Figure 1B). Whereas the western range margin in the Southern Limestone Alps is likely imposed by unsuitable siliceous bedrock (Froitzheim et al., 2008), range margins in the Northern Limestone Alps and the Dinaric Mountains are difficult to explain as no obvious geologic or topographic barriers are evident there. In addition, beech forests, the main habitat of *H. niger*, extend far beyond the species’ distribution area (Willner et al., 2009; Ellenberg and Leuschner, 2010). This strongly suggests incomplete range filling (Svenning and Skov, 2004; Willner et al., 2009; Dullinger et al., 2012a) as expected for a large-seeded species lacking adaptations to efficient vectors for dispersal over long distances and, hence, slow migration (Klotz et al., 2002).

The phylogeographic structure, inferred from analyses of genomic (RADseq; Figures 3, 4) and genetic (plastid DNA sequences; Figure 5) data, as well as from testing explicit demographic models (Figure 2; Supplementary Figure 1), suggests that *H. niger* has survived the LGM not only in the major beech forest refugium in the northwestern Balkan Peninsula (Magri et al., 2006), which reflects the species’ relatively lose connection to beech forests. Instead, survival in three disjoint refugia is supported by both genetic/genomic data and the species’ modeled distribution at the LGM (Figure 1C); two of these refugia were situated along the unglaciated margin of the Southern Limestone Alps and one, unexpectedly, was located in the eastern part of the Northern Limestone Alps.

### Long-Term Presence in Two Refugia Within the Southern Limestone Alps

The deepest genetic breaks uncovered in *H. niger* are the split between the Western and the Eastern Group in the mostly nuclear-derived RADseq data (Figure 3; Supplementary Figures 3, 5) and – in full congruence – between the Western and the Eastern Plastid Lineage in the maternally inherited plastid DNA data (Figure 5). The distribution of the western assemblages spans the Southern Limestone Alps from the species’ western range limit eastwards to the Monti Lessini east of Lago di Garda. The eastern assemblages have a much wider distribution comprising the eastern part of the Southern Limestone Alps, the northwestern Balkan Peninsula, and the Northern Limestone Alps. The SDM for the LGM (Figure 1C) supports the existence of two separate refugia at the margin of the Southern Limestone Alps and the adjacent lowlands, the first being situated close to the current western distribution limit and the second to the west of the Julian Alps; both refugia overlap with the main genetic groups (Figures 4, 5). The split between the Western and the Eastern Groups likely reflects divergent evolution in recurrent isolation in two disjoint glacial refugia. It took place in the mid-Pleistocene (ca 0.5 mya; Table 3; Supplementary Table 4), and was modeled to result from vicariance between two populations with similar initial size (expressed by parameter *s* in Table 2). Specifically, the best-fitting demographic model suggests vicariance with ancient continuous asymmetric migration, where gene flow was stronger from the Eastern to the Western Group (Figure 4; Table 2; Supplementary Table 3). This westward directionality is supported by the species’ distribution being centered in the eastern parts of the Alps (Figure 1A).

Vicariance between disjoint partial distribution areas should result in comparable levels of genetic diversity; the depth of divergence between these areas is expected to depend, among others, on the duration of isolation (e.g., Thompson, 1999; Avise, 2000; Sanmartín et al., 2001; Kropf et al., 2006). Accordingly, long-term presence in the Southern Limestone Alps – and likely in the adjacent northwestern Balkan Peninsula – is supported by high numbers of private alleles (Table 1; Figure 3C; Supplementary Figure 4) and elevated levels of nucleotide diversity (*π*; Supplementary Figure 4) in many populations from this area compared to the Northern Limestone Alps, that have diverged from the South-Eastern Subgroup relatively recently (Table 3). Within the Western Group all populations harbor a large number of private alleles indicating many local, weakly interconnected gene pools (Figure 3). In contrast, the South-Eastern Subgroup is heterogeneous; it includes populations with many private alleles both in the north and the south of the subgroup’s distribution in the Alps and the northwestern Balkan Peninsula, respectively, as well as populations with low numbers in intermittent areas (Figure 3). This may indicate the existence of two (sub)refugia within the Southeastern Group.

To date, only a few studies have unraveled refugia for forest understory species in the Southern Limestone Alps, an area previously identified as a hotspot for endemics centred in upper-montane forests (Tribsch and Schönswetter, 2003). For instance, AFLP data suggested a refugium in the southeastern Alps and the adjacent Balkan Peninsula for *Cyclamen purpurascens* Mill. (Primulaceae; Slovák et al., 2012), *Hacquetia epipactis* (Scop.) DC (Apiaceae; Urbaniak et al., 2018), and *Knautia drymeia* Heuff. (Caprifoliaceae; Rešetnik et al., 2016). The genetic split between the northwestern and the northeastern populations of *K. drymeia* largely corresponds to the main split in *H. niger* (Rešetnik et al., 2016) and suggests that similar historical events have shaped the observed phylogeographies. In a similar line, based exclusively on distribution data, Tribsch (2004) identified areas of endemism within the Southern Limestone Alps that almost perfectly match the distribution of the Western and Eastern Groups in *H. niger*.

The ranges of the Western and the Eastern Groups in the Southern Limestone Alps are likely parapatric; according to our knowledge, they are not separated by a distribution gap (Figures 1A, 3A). Previous studies have unraveled both genetic breaks paralleling discontinuities in species’ distributions (Schönswetter et al., 2002) as well as breaks running through areas of continuous habitat occupancy (Schönswetter and Tribsch, 2005; Falch et al., 2019). In spite of the absence of a distribution gap, there is little evidence for large-scale recent gene flow between the Western and the Eastern Groups. Only population 247 of the Eastern Group from the immediate contact area was slightly admixed in the STRUCTURE analysis (Figure 3A), and the best-fitting demographic models included no recent gene flow (Figure 4; Supplementary Table 3). In the absence of dedicated studies, it remains to be tested if introgression is prevented by either cross incompatibilities between the two genetic groups, local geographic barriers, or a slow migration pace due the prevalence of myrmecochory (Chrtková, 1997; Klotz et al., 2002). In contrast, the sampling gap for RADseq data within the South-Eastern Subgroup between populations 247 and 264 is an artifact explainable by the fact that in this area occurrences of *H. niger* are scarce and strictly localized (authors’ field observations).

The deep genetic divergence between the Western and the Eastern Group is not reflected in leaf shape variation; other sources of morphological variation such as flowers could not be explored due to the phenological state of the herbarium vouchers. Leaf shape was deemed an important source of characters discriminating between *H. niger* subsp. *macranthus* and the typical subspecies (Schiffner, 1889). However, in spite of exhibiting much variation (Figure 6A), only the three western-most populations were separated along the second PCA axis (Figure 6B; Supplementary Table 5). Even if one of the characters deemed diagnostic for *H. niger* subsp. *macranthus* (the distance of the widest part of the central leaflet from its tip) had the third-highest loading on the second PCA axis we suggest that –based on leaf shape variation – the traditional discrimination of two subspecies within *H. niger* is highly questionable as it does not reflect the main genetic break.

### Presence in the Northern Limestone Alps Before the LGM Supports a Northern Forest Refugium

Within the Eastern Group, populations from the Northern Limestone Alps are differentiated from those in the Southern Limestone Alps in both RADseq and plastid data sets (North-Eastern Subgroup, light blue plastid haplotype: Figures 3, 5; Supplementary Figures 3, 5), reflecting the North-South disjunction within the Eastern Alps. This differentiation – which follows the major gap in the species’ distribution that is bridged by only a handful of intermittent populations (e.g., population 436; Figure 1) – is much more shallow, however, than the differentiation between the Western and Eastern Groups within the Southern Limestone Alps.

Genetic differentiation between the North-Eastern and South-Eastern Subgroups was clearly shown by both STRUCTURE clustering and phylogenetic analyses (Figure 3C). However, genetic diversity within the North-Eastern Subgroup was low and did not support a scenario of long-term vicariance for this subgroup (Supplementary Figure 4B). Still, the increased number of private alleles in some populations of the North-Eastern Subgroup supports a shallow vicariance scenario. Based on this evidence, we conclude that the North-Eastern Subgroup cannot be considered a mere genetic subset of the South-Eastern Subgroup, as under an alternative scenario of recent dispersal (Šingliarová et al., 2008).

Vicariant isolation of the North-Eastern and South-Eastern Subgroups was further supported by explicit demographic model testing that consistently rejected recent colonization of the Northern Limestone Alps from a southern refugium. While low genetic diversity in the North-Eastern Subgroup could also be indicative for recent colonization, the best performing models always supported long-term presence in the area as well as unexpected prevalence of “ancient” (i.e., not recent) gene flow from north to south. Specifically, in the pairwise comparison of the South-Eastern and the North-Eastern Subgroups, the best-fitting model (Figure 4; Supplementary Table 3) was a vicariance scenario involving ancient continuous asymmetric migration, where gene flow from the North-Eastern to the South-Eastern Subgroup prevailed. The second-best model included an old founder event with exponential population growth of the North-Eastern Subgroup accompanied by continuous asymmetric migration from north to south. Southward range expansion of the North-Eastern subgroup is also supported by the presence of the Northern Alpine haplotype (light blue in Figure 5) in the isolated population 436 from the eastern Central Eastern Alps; unfortunately, no RADseq data are available from this population. The degree of isolation and the small size of this population renders long-term survival in that area, which was covered by ice during the LGM (van Husen, 1987), highly unlikely.

A remarkable exception from the overall good correlation between genetic relatedness and geographic proximity is presented by population 30 from the western distribution limit in the Northern Limestone Alps. This population, which grows in the vicinity of a chapel, was hypothesized to be a garden escape already during fieldwork. Its likely non-indigenous status is underlined by the presence of a plastid haplotype from the Western Plastid Lineage otherwise present in geographically distant, western-most populations in the Southern Limestone Alps (populations 229, 230, and 230; Figure 5). In contrast, the spatially close population 29, which marks the absolute western range limit of *H. niger* in the Northern Limestone Alps, seems to be autochthonous due to its occurrence in a natural habitat, i.e., undisturbed forest vegetation in a deep gorge with difficult access, and the presence of the “correct” plastid haplotype. While an anthropogenic introduction of population 29 from eastern parts of the Northern Limestone Alps cannot be fully rejected given this evidence, a long-distance dispersal event explaining the haplotype of population 30 seems highly unlikely.

The split between the North-Eastern and the South-Eastern Subgroup was dated to ca 0.1 mya employing the BPP approach and the RADseq data, which is well before the LGM (Table 3; Supplementary Table 4). This clearly rejects the hypothesis that the partial distribution area in the Northern Limestone Alps is the result of postglacial colonization. Instead, the North-Eastern Subgroup likely reached this area during the last interglacial and became genetically divergent from its ancestral population due to isolation in a refugium. A massive demographic contraction in the subsequent Last Glacial Period likely caused the observed low genetic diversity and, in accordance with the niche modeling, also points at a lack of large-scale refugia in this area (Figures 1C, 3A). In the same line, the plastid data reject postglacial colonization. The exclusively occurring plastid haplotype (coded light blue in Figure 5) is restricted to the Northern Limestone Alps. Evolution of a new plastid DNA haplotype separated by a single mutational step within the last 20 kya would require a mutation rate of 0.03 mutations per site per mya, which is exceeding any published rate for plastid DNA (Wolfe et al., 1987; Muse, 2000; Willyard et al., 2007). However, in spite of suggesting presence of *H. niger* in the Northern Limestone Alps during the LGM, both the presence of a single, yet endemic plastid haplotype and the low number of private alleles across all populations underline the relatively recent origin of these populations (Table 1; Figure 3; Supplementary Table 1; Supplementary Figure 4). A glacial forest refugium in the eastern-most Northern Limestone Alps is strongly supported by the presence of several regional endemics (Niklfeld, 1972; Tribsch and Schönswetter, 2003; Rabitsch and Essl, 2009), among others *Callianthemum anemonoides* (Kadereit et al., 2019) and the genetically strongly isolated *Euphorbia saxatilis* (Frajman and Schönswetter, 2017).

Two aspects of the spatiotemporal diversification of *H. niger* remain difficult to explain. First, the contrasting depth of genetic splits (Figure 3C) leaves no doubt that the Northern Limestone Alps were colonized from the south. However, demographic models (Table 2) suggest a predominance of gene flow after this divergence in the opposite direction. This is also supported by the admixed status of the northern-most populations of the South-Eastern Subgroup in the STRUCTURE analysis conducted within the Eastern Group (Figure 3B). This gene flow into the Southern Limestone Alps must have occurred *via* pollen as it is seen only in the biparentally inherited RADseq data but not in the maternally transmitted plastid haplotypes (Figures 3B, 5). Second, the projected SDM for the LGM (Figure 1C) does not indicate the presence of climatically suitable areas in the Northern Limestone Alps in spite of the unambiguous evidence from the genetic data. However, since the SDMs have a rather coarse resolution of 1 km, spatially restricted sites with suitable microclimate could have been present even in areas predicted as unsuitable by our model (Randin et al., 2009). Moreover, LGM climate data have an even coarser spatial resolution of 2.5°, and fine-scale climatic variability is largely unknown. Thus, local climatic conditions along the northeastern margin of the Alps might have been less extreme than suggested by our downscaled data.

Finally, what do the range dynamics of *H. niger* presented here actually tell us about the forest vegetation of the Northern Limestone Alps during the LGM? In present-day vegetation, *H. niger* prefers montane beech forests and mixed stands dominated by spruce, fir, and beech (Supplementary Table 6). However, the species also occurs in subalpine forests dominated by larch and pine species, the same trees that have formed a discontinuous belt along the southern margin of the Alps and adjacent regions during the LGM (Monegato et al., 2015). A similar vegetation type was probably present in the eastern part of the Northern Limestone Alps, albeit in more restricted areas than in the Southern Limestone Alps. We speculate that the current preference for deciduous forests dominated by beech might be explained by weaker competition in the relatively sparse herb layer as compared to the dense understory of open pine forests, which is usually dominated by dwarf shrubs of the Ericaceae family (Willner and Grabherr, 2007; Ellenberg and Leuschner, 2010). We suggest, therefore, that *H. niger* survived the LGM in open coniferous forests at the southern and northeastern margin of the Alps as well as in the northwestern Balkan Peninsula. Being a slow migrator, the species has likely survived repeated glacial-interglacial circles in distributional stasis while the composition of the tree canopy changed in the meanwhile.

## Data Availability Statement

The datasets presented in this study can be found in online repositories. The names of the repository/repositories and accession number(s) can be found at: https://www.ncbi.nlm.nih.gov/genbank/, MW755200-MW755299, https://www.ncbi.nlm.nih.gov/, BioProject PRJNA716097, accessions numbers SRR14023579–SRR14023696, SRR14181234–SRR14181236, and SRR14181231–SRR14181233.

## Author Contributions

PS, CD, EZ, KH, and WW conceived the study. CD and many other colleagues (listed in the acknowledgments) collected the samples. JW, WW, AG, and KH compiled the distribution and climatic data and produced the species distribution models. EZ and DL carried out the molecular lab work. EZ and PK analyzed the genomic data. BF analyzed the genetic data. CD produced and analyzed the morphometric data. EZ and PS wrote major parts of the manuscript, with exception of the parts about SDMs written by KH. All authors read and edited the final version of the manuscript.

### Conflict of Interest

The authors declare that the research was conducted in the absence of any commercial or financial relationships that could be construed as a potential conflict of interest.

## Footnotes

^1^http://sweetgum.nybg.org/science/ih/

^2^https://www.cbd.int/abs/about

^3^http://www.vbcf.ac.at/ngs/

^4^https://github.com/dportik/dadi_pipeline

^5^https://github.com/isaacovercast/easySFS

^6^https://www.eea.europa.eu/data-and-maps/data/copernicus-land-monitoring-service-eu-dem

## Supplementary Material

The Supplementary Material for this article can be found online at:

https://www.frontiersin.org/articles/10.3389/fpls.2021.683043/full#supplementary-material
