# Supplementary material for: Evidence for Glacial Refugia of the Forest Understorey Species Helleborus niger (Ranunculaceae) in the Southern as Well as in the Northern Limestone Alps
Source: Front Plant Sci. 2021 May 10;12:683043. doi: 10.3389/fpls.2021.683043 (PMC8141911; doi:10.3389/fpls.2021.683043)

**Supporting Information**

**Supplementary Table 1**. Locality details for sampled populations of *Helleborus niger* and the outgroups (ID, identifier used in this paper; Lab ID, identifier used in GenBank; Latitude North in decimal degrees, Longitude East in decimal degrees, ID of the herbarium voucher stored at the herbarium IB of the University of Innsbruck); Short Read Archive (SRA) and Genbank accession numbers for the RADseq data (SRA RAD; BioProject PRJNA716097, only the last three digits of accession numbers starting with ‘SAMN18394…’ are given; other accession numbers are given fully) and the plastid *rpoB–psbM* region (Plastid DNA), respectively; inclusion of a population in the morphometric analysis (Morpho); genetic group based on STRUCTURE analyses (RADseq group: NE, North-Eastern Subgroup; SE, South-Eastern Subgroup; W, Western Group) and population genetic descriptors based on the RADseq data.

| **ID** | **Lab ID** | **Latitude N** | **Longitude E** | **Herbarium ID** | **SRA RAD**  SAMN18394… | **Plastid DNA** | **Morpho** | **RADseq group** | **Private alleles** | **Sites recovered** | **% Polymorphic Loci** | **Nucleotide diversity (Pi)** |
| --- | --- | --- | --- | --- | --- | --- | --- | --- | --- | --- | --- | --- |
| 29 | 53 | 47.277778 | 11.327500 | IB 110360 | - | MW755206, MW755256 | 0 | - | - | - | - | - |
| 30 | 54 | 47.283333 | 11.365833 | IB 110361 | - | MW755207, MW755257 | 0 | - | - | - | - | - |
| 35 | 41 | 47.588739 | 12.186911 | IB 110350 | - | MW755237, MW755287 | 1 | - | - | - | - | - |
| 41 | 38 | 47.714761 | 13.551689 | IB 110347 | 696–698 | MW755234, MW755284 | 1 | NE | 1 | 76023 | 0.1565 | 0.0008 |
| 44 | 37 | 47.398589 | 13.609269 | IB 110346 | 693–695 | MW755233, MW755283 | 1 | NE | 3 | 62058 | 0.137 | 0.0007 |
| 47 | 39 | 47.823669 | 14.121939 | IB 110348 | 699–701 | MW755235, MW755285 | 1 | NE | 1 | 66236 | 0.1555 | 0.0008 |
| 50 | 40 | 47.870061 | 14.610569 | IB 110349 | 702–703 | MW755236, MW755286 | 1 | NE | 0 | 88863 | 0.1395 | 0.001 |
| 53 | 32 | 47.528500 | 15.064889 | IB 110341 | 690–692 | MW755228, MW755278 | 1 | NE | 2 | 77357 | 0.203 | 0.001 |
| 58 | 29 | 47.975769 | 15.203069 | IB 110338 | 681–683 | MW755225, MW755275 | 1 | NE | 0 | 76651 | 0.1866 | 0.001 |
| 61 | 31 | 47.672811 | 15.457211 | IB 110340 | 687–689 | MW755227, MW755277 | 1 | NE | 2 | 72912 | 0.1865 | 0.001 |
| 64 | 30 | 47.960589 | 15.531261 | IB 110339 | 684–686 | MW755226, MW755276 | 1 | NE | 3 | 72984 | 0.174 | 0.0009 |
| 67 | 28 | 47.878339 | 15.811561 | IB 110337 | 678–680 | MW755224, MW755274 | 1 | NE | 1 | 62578 | 0.155 | 0.0008 |
| 229 | 15 | 45.966369 | 8.878831 | IB 110324 | 601–603 | MW755212, MW755262 | 1 | W | 11 | 56720 | 0.2292 | 0.0012 |
| 230 | 16 | 46.038831 | 9.010739 | IB 110325 | 604–606 | MW755213, MW755263 | 1 | W | 9 | 59756 | 0.2376 | 0.0013 |
| 232 | 14 | 45.931589 | 9.496289 | IB 110323 | 598–600 | MW755211, MW755261 | 1 | W | 9 | 64906 | 0.2696 | 0.0014 |
| 234 | 12 | 45.754000 | 9.809269 | IB 110321 | 592–594 | MW755209, MW755259 | 1 | W | 11 | 67224 | 0.2841 | 0.0015 |
| 235 | 13 | 45.971589 | 9.964239 | IB 110322 | 595–597 | MW755210, MW755260 | 1 | W | 13 | 61347 | 0.2543 | 0.0013 |
| 236 | 48 | 45.695900 | 10.089219 | IB 110357 | 666–668 | MW755244, MW755294 | 0 | - | - | - | - | - |
| 239 | 11 | 45.771489 | 10.486569 | IB 110320 | 589–591 | MW755208, MW755258 | 1 | W | 16 | 58947 | 0.2969 | 0.0016 |
| 240 | 10 | 46.073781 | 10.769931 | IB 110319 | 586–588 | MW755201, MW755251 | 1 | W | 12 | 54586 | 0.2235 | 0.0011 |
| 243 | 9 | 45.758000 | 11.188439 | IB 110365 | 609–611 | MW755249, MW755299 | 1 | W | 11 | 60634 | 0.2062 | 0.0011 |
| 244 | 8 | 45.874100 | 11.436650 | IB 110364 | 607–608 | MW755248, MW755298 | 1 | W | 15 | 81390 | 0.1794 | 0.0012 |
| 247 | 7 | 45.934339 | 11.834211 | IB 110363 | 675–677 | MW755247, MW755297 | 1 | SE | 7 | 75574 | 0.2144 | 0.0011 |
| 253 | 51 | 46.193000 | 12.552000 | IB 110367 | - | MW755204, MW755254 | 0 | - | - | - | - | - |
| 255 | 50 | 46.326000 | 12.793000 | IB 110366 | - | MW755203, MW755253 | 0 | - | - | - | - | - |
| 259 | 52 | 46.192000 | 12.970000 | IB 110368 | - | MW755205, MW755255 | 0 | - | - | - | - | - |
| 264 | 6 | 46.506150 | 13.265961 | IB 110362 | 672–674 | MW755246, MW755296 | 1 | SE | 12 | 78713 | 0.2249 | 0.0012 |
| 265 | 36 | 46.705481 | 13.416561 | IB 110345 | 651–653 | MW755232, MW755282 | 1 | SE | 1 | 71119 | 0.1758 | 0.0009 |
| 267 | 5 | 46.471500 | 13.568119 | IB 110359 | 669–671 | MW755245, MW755295 | 1 | SE | 6 | 77097 | 0.2192 | 0.0011 |
| 272 | 35 | 46.475039 | 13.781200 | IB 110344 | 648–650 | MW755231, MW755281 | 1 | SE | 4 | 77005 | 0.1948 | 0.001 |
| 273 | 43 | 46.184800 | 13.717200 | IB 110352 | 657–659 | MW755239, MW755289 | 1 | SE | 1 | 51637 | 0.1356 | 0.0007 |
| 283 | 46 | 45.894181 | 13.988000 | IB 110355 | SRR14181234–SRR14181236 | MW755242, MW755292 | 1 | SE | 2 | 64793 | 0.2006 | 0.001 |
| 286 | 44 | 46.196269 | 14.270350 | IB 110353 | 660–662 | MW755240, MW755290 | 1 | SE | 2 | 52623 | 0.1406 | 0.0007 |
| 288 | 34 | 46.397850 | 14.338761 | IB 110343 | 645–647 | MW755230, MW755280 | 1 | SE | 4 | 71758 | 0.1547 | 0.0008 |
| 295 | 33 | 46.470450 | 14.787350 | IB 110342 | 642–644 | MW755229, MW755279 | 1 | SE | 6 | 75117 | 0.1984 | 0.001 |
| 299 | 45 | 45.802900 | 14.376569 | IB 110354 | 663–665 | MW755241, MW755291 | 1 | SE | 4 | 83954 | 0.212 | 0.0011 |
| 305 | 47 | 46.033000 | 15.624000 | IB 110356 | - | MW755243, MW755293 | 1 | - | - | - | - | - |
| 318 | 19 | 45.778542 | 15.650369 | IB 110328 | 618–620 | MW755216, MW755266 | 1 | SE | 10 | 80117 | 0.2234 | 0.0011 |
| 320 | 42 | 45.682000 | 15.068000 | IB 110351 | 654–656 | MW755238, MW755288 | 1 | SE | 3 | 65435 | 0.1712 | 0.0009 |
| 325 | 20 | 45.462425 | 14.552592 | IB 110329 | 621–623 | MW755217, MW755267 | 1 | SE | 8 | 78784 | 0.1993 | 0.001 |
| 330 | 26 | 45.414947 | 14.622653 | IB 110335 | 639–641 | MW755223, MW755273 | 1 | SE | 2 | 72904 | 0.1838 | 0.001 |
| 332 | 18 | 45.414947 | 14.622653 | IB 110327 | 615–617 | MW755215, MW755265 | 1 | SE | 3 | 81002 | 0.2333 | 0.0012 |
| 333 | 24 | 45.486308 | 14.767264 | IB 110333 | 633–635 | MW755221, MW755271 | 1 | SE | 2 | 81628 | 0.2009 | 0.001 |
| 339 | 23 | 45.446294 | 14.875033 | IB 110332 | 630–632 | MW755220, MW755270 | 1 | SE | 1 | 59379 | 0.1701 | 0.0009 |
| 346 | 17 | 45.251286 | 15.164300 | IB 110326 | 612–614 | MW755214, MW755264 | 1 | SE | 10 | 78339 | 0.2374 | 0.0012 |
| 352 | 25 | 45.220864 | 15.244814 | IB 110334 | 636–638 | MW755222, MW755272 | 1 | SE | 3 | 78963 | 0.2178 | 0.0011 |
| 359 | 22 | 44.929425 | 15.555033 | IB 110331 | 627–629 | MW755219, MW755269 | 1 | SE | 2 | 63207 | 0.1804 | 0.001 |
| 361 | 21 | 44.830672 | 15.554764 | IB 110330 | 624–626 | MW755218, MW755268 | 1 | SE | 6 | 80209 | 0.2232 | 0.0011 |
| 436 | 49 | 46.968889 | 13.946944 | IB 110358 | - | MW755202, MW755252 | 1 | - | - | - | - | - |
| *H. foetidus* 1 |  |  |  |  | SRR14181232, SRR14181233 | MW755200, MW755250 |  |  | - | - | - | - |
| *H. foetidus* 2 |  |  |  |  | SRR14181231 |  |  |  | - | - | - | - |

**Supplementary Table 2**. Characteristics of the different RADseq datasets used in various analyses presented, including number of investigated individuals (N_ind_), populations (N_pop_) and variants (N_variants_).

| **Analysis** | N_ind_ | N_pop_ | N_variants_ |
| --- | --- | --- | --- |
| RaxML (incl. outgroup) | 121 | 41 | 4914 |
| STRUCTURE - Eastern and Western Group | 118 | 40 | 1074 |
| STRUCTURE - Eastern Group | 92 | 31 | 1028 |
| Dadi - Western Group & South-Eastern Subgroup | 12/14 | 2 | 802/700 |
| Dadi - South-Eastern and North-Eastern Subgroup | 12/12 | 2 | 430/281 |
| BPP | 29 | 19 | 900 |

**Supplementary Table 3.** Results of all 2D demographic models tested for pairwise population comparisons between (i) Eastern vs. Western Group, (ii) Western vs Eastern Group and (iii) South-Eastern vs North-Eastern Subgroup. All models tested are visualised and provided with full and abbreviated names in Supplementary Fig. 1. Other abbreviations are as follows: AIC, Akaike information criterion; ΔAIC, difference in AIC to the best-scoring model of the comparison of two particular groups; ω_i_, Akaike weight (calculated over all models for the comparisons of Eastern vs. Western Group, and Western vs. Eastern Group); theta, the effective mutation rate of the reference population (fully defined in the caption of Table 2).

| **Eastern vs. Western Group** |  |  |  |  |  |  |
| --- | --- | --- | --- | --- | --- | --- |
| Model | Log-likelihood | AIC | ΔAIC | ω_i_ | Chi-squared | Theta |
| **vic_anc_asym_mig** | **-257.13** | **530.26** | **0** | **0.813** | **169.030** | **515.29** |
| vic_no_mig | -270.09 | 550.18 | 19.92 | 0.000 | 205.640 | 361.74 |
| founder_nomig | -278.38 | 566.76 | 36.5 | 0.000 | 240.780 | 152.6 |
| founder_no_mig_two_epoch | -277.55 | 567.1 | 36.84 | 0.000 | 241.050 | 73.15 |
| founder_anc_asym_two_epoch | -277.91 | 571.82 | 41.56 | 0.000 | 244.770 | 19.08 |
| founder_asym | -278.71 | 571.42 | 41.16 | 0.000 | 243.890 | 2772.08 |
| founder_sec_contact_asym_two_epoch | -278.96 | 573.92 | 43.66 | 0.000 | 249.060 | 96.45 |
| vic_sec_contact_asym_mig | -305.43 | 626.86 | 96.6 | 0.000 | 288.810 | 2183.49 |
|  |  |  |  |  |  |  |
| **Western vs. Eastern Group** |  |  |  |  |  |  |
| Model | Log-likelihood | AIC | ΔAIC | ω_i_ | Chi-squared | Theta |
| vic_no_mig | -262.21 | 534.42 | 4.16 | 0.102 | 183.990 | 369.470 |
| vic_anc_asym_mig | -259.78 | 535.56 | 5.3 | 0.057 | 181.380 | 1077.330 |
| founder_anc_asym_two_epoch | -260.54 | 537.08 | 6.82 | 0.027 | 181.470 | 2420.740 |
| founder_nomig | -267.41 | 544.82 | 14.56 | 0.001 | 204.070 | 203.690 |
| founder_no_mig_two_epoch | -266.24 | 544.48 | 14.22 | 0.001 | 202.140 | 290.250 |
| founder_asym | -269.9 | 553.8 | 23.54 | 0.000 | 213.690 | 38.570 |
| founder_sec_contact_asym_two_epoch | -304.42 | 624.84 | 94.58 | 0.000 | 295.490 | 2117.900 |
| vic_sec_contact_asym_mig | -307.28 | 630.56 | 100.3 | 0.000 | 288.620 | 1577.65 |
|  |  |  |  |  |  |  |
| **South-Eastern vs North-Eastern Subgroup** |  |  |  |  |  |  |
| Model | Log-likelihood | AIC | ΔAIC | ω_i_ | Chi-squared | Theta |
| **vic_anc_asym_mig** | **-139.85** | **295.7** | **0** | **0.512** | **268.540** | **123.840** |
| founder_anc_asym_two_epoch | -140.49 | 296.98 | 1.28 | 0.270 | 236.440 | 23.870 |
| founder_sec_contact_asym_two_epoch | -140.96 | 297.92 | 2.22 | 0.169 | 494.420 | 179.190 |
| vic_no_mig | -145.3 | 300.6 | 4.9 | 0.044 | 122.210 | 109.600 |
| vic_sec_contact_asym_mig | -144.68 | 305.36 | 9.66 | 0.004 | 147.660 | 107.780 |
| founder_asym | -148.61 | 311.22 | 15.52 | 0.000 | 610.300 | 54.34 |
| founder_nomig | -158.88 | 327.76 | 32.06 | 0.000 | 113.110 | 708.38 |
| founder_no_mig_two_epoch | -158.31 | 328.62 | 32.92 | 0.000 | 106.760 | 32.24 |

**Supplementary Table 4.** Mean τ values (τ = 2µt; µ = mutation rate per site per generation, t = divergence time) and lower and upper limit of highest posterior density (HPD, 95% credibility) estimated via the multi-species coalescent model implemented in BPP. Values are given for five randomly selected subsets of RADseq tags.

|  | **Western and Eastern Group** | | | **South-Eastern and North-Eastern Subgroup** | | |
| --- | --- | --- | --- | --- | --- | --- |
| **N RADseq tags** | **Mean τ** | **lower HPD** | **upper HPD** | **Mean τ** | **lower HPD** | **upper HPD** |
| 100 | 6.79E-04 | 4.42E-04 | 9.53E-04 | 2.30E-04 | 1.05E-04 | 3.63E-04 |
| 200 | 5.23E-04 | 3.48E-04 | 6.90E-04 | 1.49E-04 | 8.50E-05 | 2.17E-04 |
| 300 | 5.49E-04 | 3.91E-04 | 6.82E-04 | 1.34E-04 | 8.00E-05 | 1.93E-04 |
| 400 | 5.60E-04 | 4.56E-04 | 6.58E-04 | 1.36E-04 | 8.50E-05 | 1.86E-04 |
| 500 | 6.20E-04 | 4.86E-04 | 7.70E-04 | 1.17E-04 | 7.10E-05 | 1.67E-04 |

**Supplementary Table 5.** Canonical structure showing the correlation of 15 morphological characters describing the shape of rosette leaves of *Helleborus niger* with the canonical axis. The highest values are in bold.

| **character** | **correlation** |
| --- | --- |
| 1 | **0.3125** |
| 2 | **0.7156** |
| 3 | -0.1264 |
| 4 | 0.0014 |
| 5 | 0.2087 |
| 6 | **0.5967** |
| 7 | -0.2127 |
| 8 | 0.1994 |
| 9 | 0.0524 |
| 10 | 0.2882 |
| 11 | 0.1237 |
| 12 | 0.1092 |
| 13 | 0.05517 |
| 14 | -0.0697 |
| 15 | -0.3086 |

**Supplementary Table 6.** Percentage frequency of *Helleborus niger* in forest communities on calcareous soils in the eastern part of the Northern Limestone Alps (Lower Austria, Upper Austria, Styria). Classification of vegetation plots into associations follows Willner and Grabherr (2007)*. 1: Adenostylo glabrae-Fagetum, 2: Saxifrago rotundifoliae-Fagetum, 3: Adenostylo glabrae-Piceetum, 4: Erico-Pinetum sylvestris, 5: Rododendro hirsuti-Pinetum cembrae, 6: Rhodothamno-Laricetum, 7: Erico-Pinetum prostratae and Rhododendro hirsuti-Pinetum prostratae. In the lower part of the table, the frequency of the dominant woody species is given. The dominant species is indicated in bold.

| **Association** | **1** | **2** | **3** | **4** | **5** | **6** | **7** |
| --- | --- | --- | --- | --- | --- | --- | --- |
| Number of plots | 280 | 129 | 88 | 110 | 24 | 46 | 191 |
| *Helleborus niger* | 60 | 83 | 48 | 45 | 13 | 37 | 18 |
| *Fagus sylvatica* | **100** | **100** | 14 | 11 | 4 | 20 | 1 |
| *Picea abies* | 94 | 90 | **100** | 68 | 54 | 85 | 14 |
| *Pinus sylvestris* | 13 | 1 | 5 | **100** | 0 | 4 | 2 |
| *Pinus cembra* | 0 | 2 | 2 | 0 | **100** | 0 | 3 |
| *Larix decidua* | 40 | 42 | 65 | 20 | 92 | **100** | 15 |
| *Pinus mugo* | 2 | 5 | 18 | 11 | 83 | 70 | **100** |

*Willner, W., and Grabherr, G. (2007). Die Wälder und Gebüsche Österreichs. Heidelberg: Elsevier.

**Supplementary figures**

**Supplementary Fig. 1**. Models used for demographic modeling. Framed models were designed for this study, unframed models were taken from Charles et al. (2018).


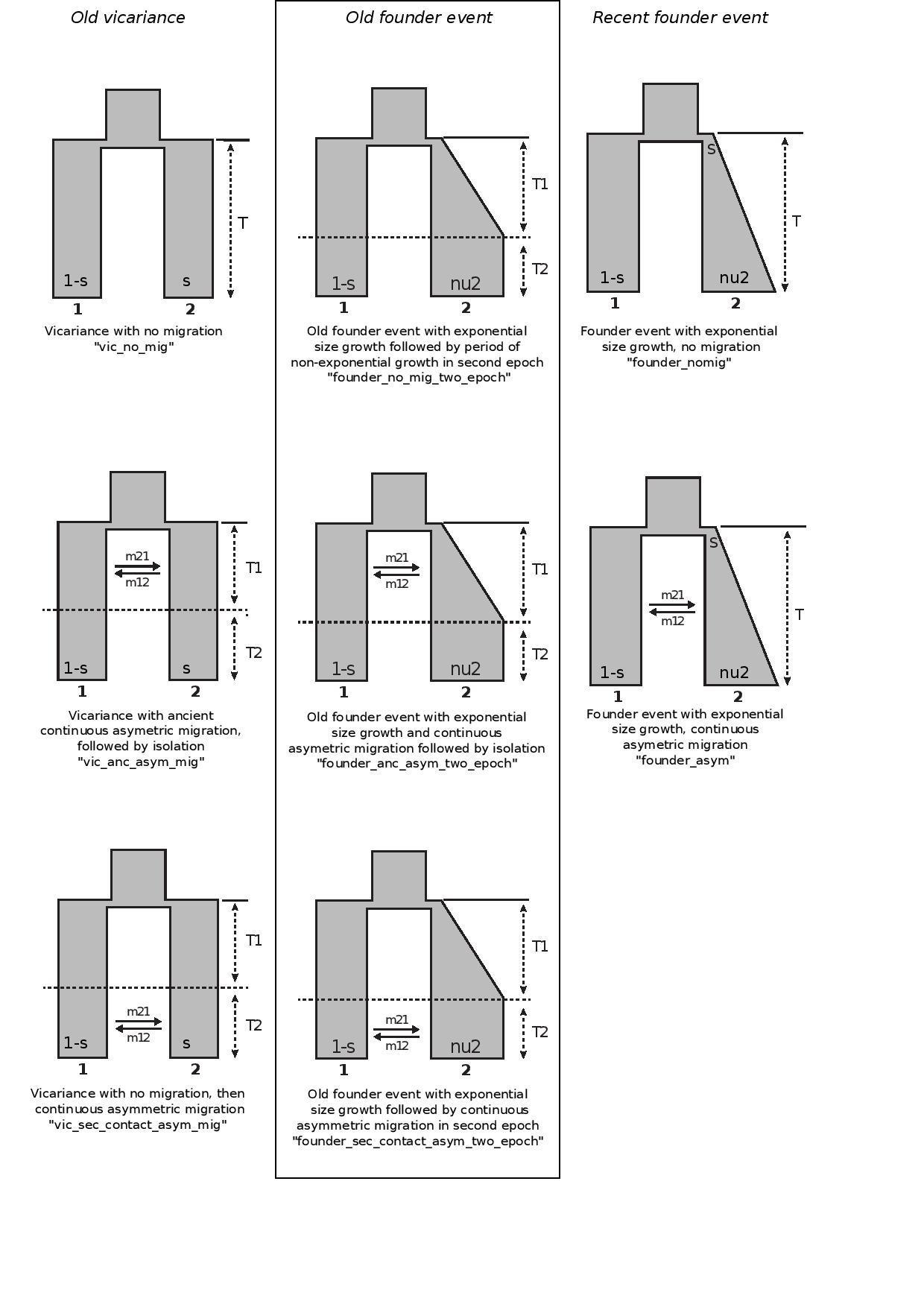


**Supplementary Fig. 2.** Potential distribution of *Helleborus niger* at the Younger Dryas 12,100 BP derived from a SDM parameterized with current climatic conditions only. The occurrence probability derived from SDM is indicated by a colour shade ramp; the terrestrial surface with occurrence probability < 0.02 is in grey. The current distribution is indicated by black lines.


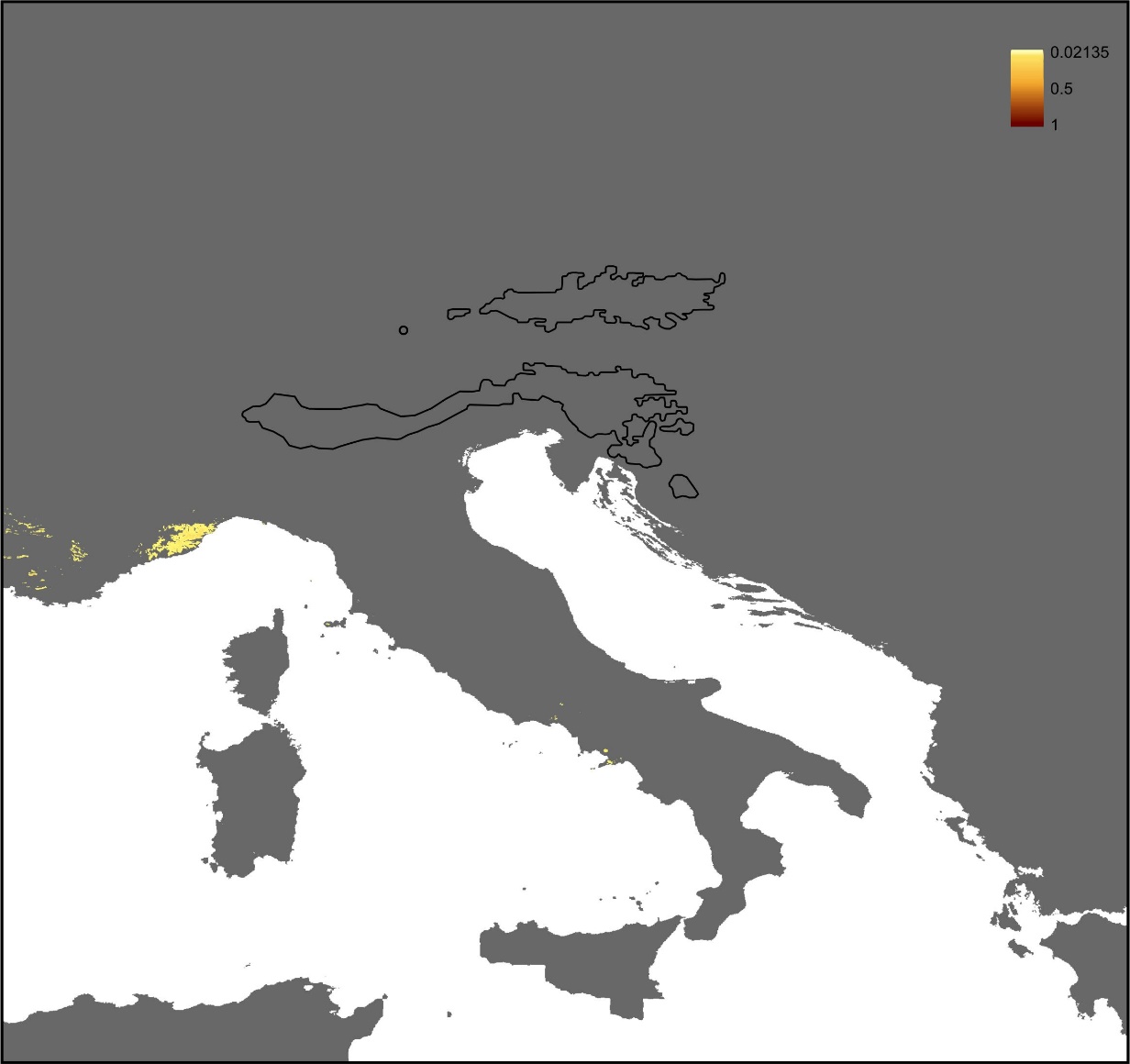


**Supplementary Fig. 3.** Summary statistics of STRUCTURE⁠ analyses of the two datasets of *H. niger*, the complete dataset (upper row) and the dataset including only the Eastern Group (lower row). Values of Ln probability of the model for each number of groups (K) are plotted against K values in the first panel, and delta K value coefficients among runs are plotted against K values in the second panel.

**
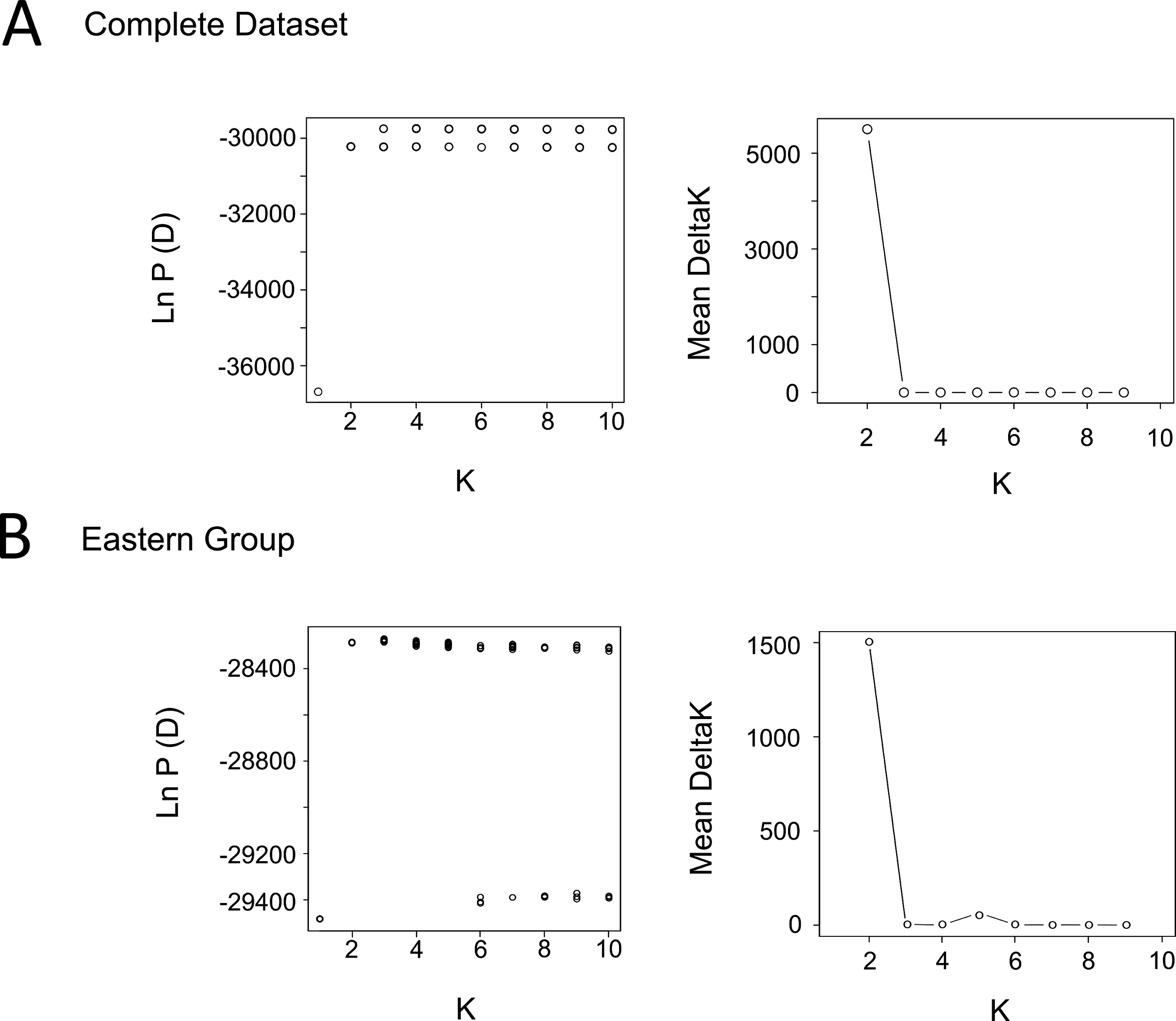
**

**Supplementary Fig. 4**. Comparison of the number of private alleles and the nucleotide diversity (π) per population across the three phylogeographic groups identified in *Helleborus niger* based on RADseq. Results of Mann-Whitney significance tests of pairwise differences in distributions between groups are indicated by asterisks (***, p-value < 0.001; **, p < 0.05).

**
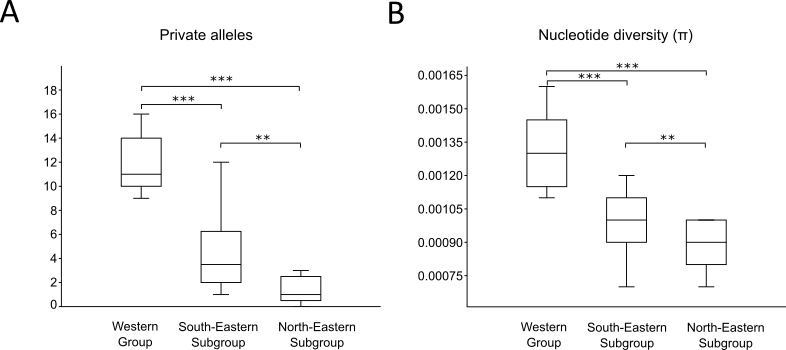
**

**Supplementary Fig. 5.** RADseq-based RaxML phylogeny of *Helleborus niger*; the tree was rooted with *H. foetidus*. Numbers above branches are bootstrap values, population IDs ranging from 5 to 48 correspond to Supplementary Table 1 and are followed by one digit representing the sequenced individual.


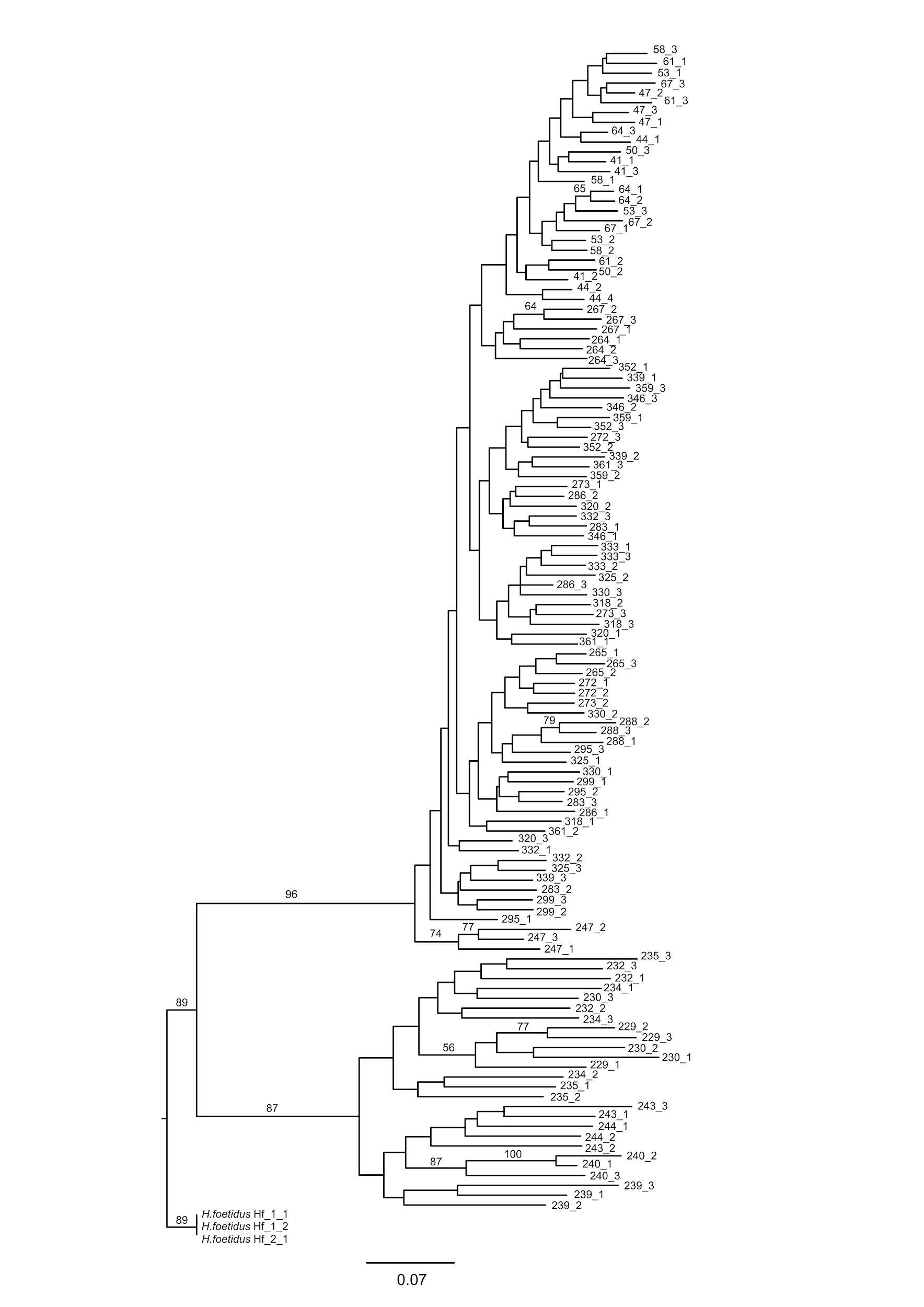

Supplement: Supplementary file 1 [file Data_Sheet_1.docx]
